# Recent Progress on Carbon Quantum Dots Based Photocatalysis

**DOI:** 10.3389/fchem.2022.881495

**Published:** 2022-04-25

**Authors:** Hwapyung Jung, Vijay S. Sapner, Arindam Adhikari, Bhaskar R. Sathe, Rajkumar Patel

**Affiliations:** ^1^ Nano Science and Engineering, Integrated Science and Engineering Division (ISED), Underwood International College, Yonsei University, Incheon, South Korea; ^2^ Department of Chemistry, Dr. Babasaheb Ambedkar Marathwada University Aurangabad, Seoul, South Korea; ^3^ Aadarsh Innovations, Pune, India; ^4^ Energy and Environmental Science and Engineering (EESE), Integrated Science and Engineering Division (ISED), Underwood International College, Yonsei University, Incheon, South Korea

**Keywords:** carbon quantum dots (CQDs), photocatalysis, modified quantum dots, composite quantum dots, energy and environmental remediation, photovoltaic devices

## Abstract

As a novel carbon allotrope, carbon quantum dots (CQDs) have been investigated in various fields, including photocatalysis, bioimaging, optoelectronics, energy and photovoltaic devices, biosensing, and drug delivery owing to their unique optical and electronic properties. In particular, CQDs’ excellent sunlight harvesting ability, tunable photoluminescence (PL), up-conversion photoluminescence (UCPL), and efficient photo-excited electron transfer have enabled their applications in photocatalysis. This work focuses on the recent progress on CQDs-related materials’ synthesis, properties, and applications in photocatalysis.

## Graphical Abstract

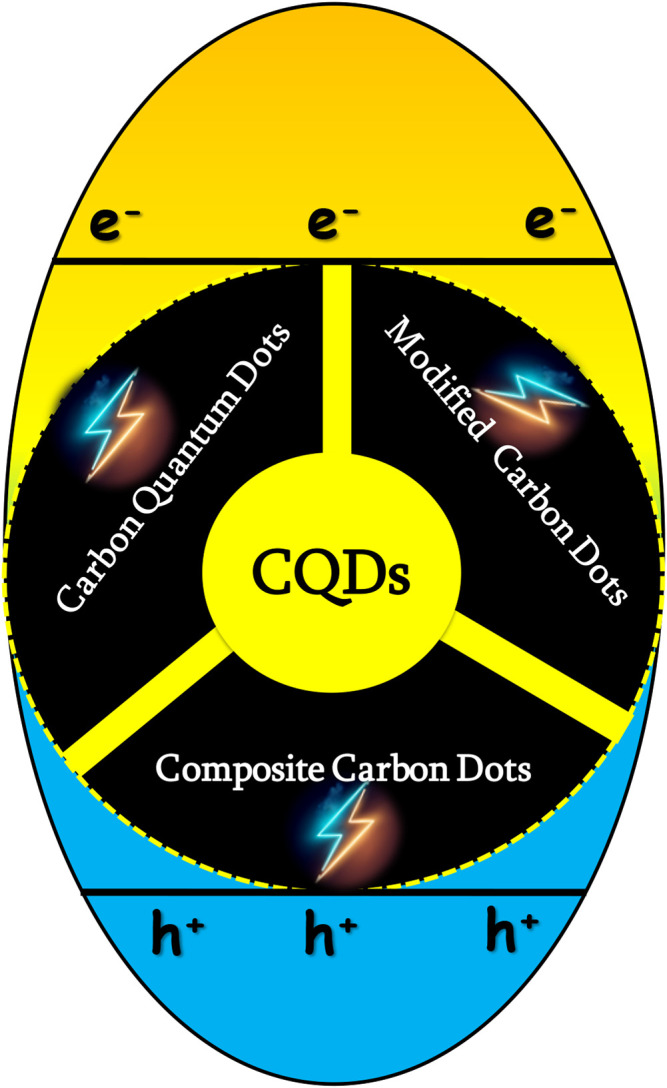

## 1 Introduction

A new kind of carbon allotrope, carbon quantum dots (CQDs), carbon dots (CDs, C-dots, or C_Dots_), or carbon nanodots (Liu et al., 2016) are quasi-spherical, monodisperse carbon nanoparticles with a diameter below 10 nm. Graphene quantum dots (GQDs) are a kind of CQDs with relatively high crystallinity over other allotropic forms (Li et al., 2015). CQDs possess both electronic properties of carbon materials and optical properties of quantum dots (Yu et al., 2016). They were discovered in 2004 (Xu et al., 2004) and got their name in 2006 (Sun et al., 2006). CQDs have been researched in various fields such as photocatalysis (Hu et al., 2013a; Han et al., 2013; Hou et al., 2015; Fang et al., 2016), bioimaging (Ray et al., 2009; Antaris et al., 2013; Bhunia et al., 2013), optoelectronics (Guo et al., 2012; Chen Q.-L. et al., 2013; Bourlinos et al., 2013; Ma et al., 2015), photovoltaic devices (Zhang et al., 2013; Suzuki et al., 2015), biosensing (Kleinauskas et al., 2013; Zhu et al., 2013), and drug delivery (Tang J. et al., 2013; Feng et al., 2016).

CQDs have an amorphous or nanocrystalline core, which is mainly sp^2^ carbon, with the lattice spacings of graphite and oxygenic functional groups (5–50 wt%) on the surface, which give water solubility and the possibility of further functionalization (Yang et al., 2009a; Yang et al., 2009b; Baker and Baker, 2010). This structure gives CQDs having unique properties, including excellent sunlight harvesting ability, tunable photoluminescence (PL), up-converted photoluminescence (UCPL), and efficient photo-excited electron transfer. Depending on synthesis methods, functional groups on the surface can be modified to further tune the PL of CQDs. By introducing electron donor and/or electron acceptor, PL of CQDs can be quenched (Wang et al., 2009; Basit et al., 2022; Chang et al., 2022).

**FIGURE 1:**
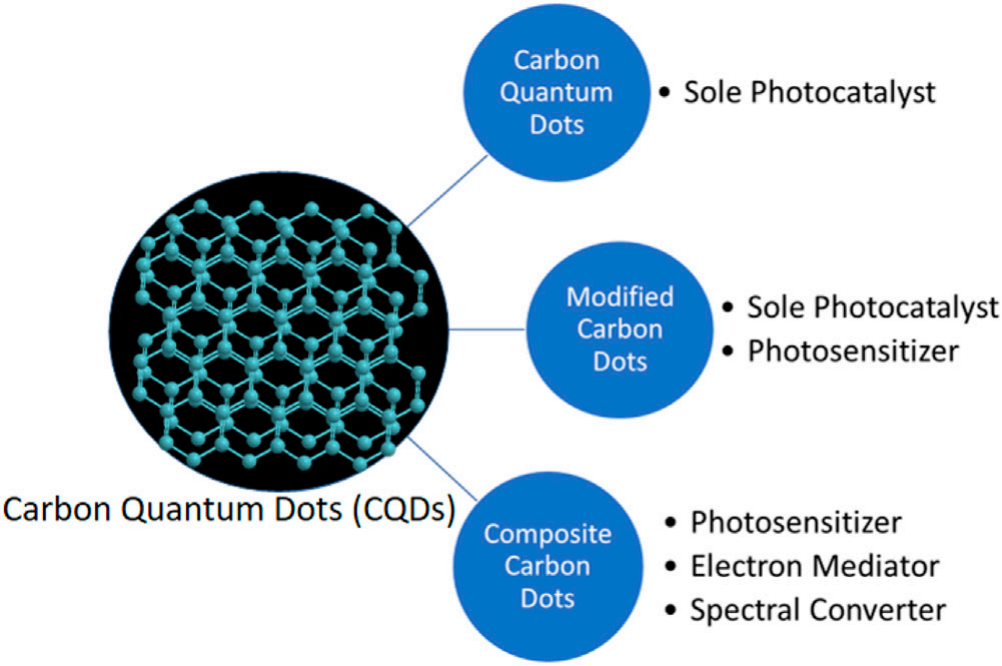
Schematic overview of applications of CQDs-related materials as sole photocatalysts, photosensitizers, electron mediators, and spectral converters.

The unique properties of CQDs enable their applications in photocatalysis (Murali et al., 2021; Syed et al., 2021; Yao et al., 2022), where photogenerated electrons and holes do the job. The UCPL of CQDs makes the use of the full spectrum of solar light possible to increase the light absorption and thus the photogenerated electrons and holes (Wang and Hu, 2014; Farshbaf et al., 2018; An et al., 2022; Preethi et al., 2022; Saeidi et al., 2022; Su et al., 2022). The efficient photo-excited electron transfer of CQDs retard the recombination of electron-hole pairs to increase the lifetime of photogenerated electrons and holes (Liang et al., 2021; Liu et al., 2021; Mahmood et al., 2021). Hence, CQDs can be used in photocatalysis. CQDs can act as a sole photocatalyst or enhance the photocatalytic activity of other photocatalysts as an electron mediator, a photosensitizer, and/or a spectral converter (Figure 1).

This work focuses on recent progress on CQDs-related materials’ synthesis, properties, and applications in photocatalysis. Subsequently, Sections 2–4 will be about carbon quantum dots, modified carbon dots, and composite carbon dots, respectively. In each section, the synthesis methods, characterization, and photocatalysis of corresponding materials will be introduced. The synthesis of those materials and the pollutants they photodegrade are summarized in Table 1, and Section 5 will present the conclusion.

**TABLE 1:** CQDs-based materials’ synthesis and application in photocatalytic pollution control processes.

CQDs catalytic system	Source	Method	Pollutants	CQDs	References
Graphene quantum dots	Citric acid	Pyrolysis	New fuchsin dye	Sole Photocatalyst Photosensitizer	(Roushani et al., 2015)
Carbon dots	*Elettariacardamomum*	Solution extraction	Congo red and methylene blue dyes		(Zaib et al., 2021)
Carbon dots	Bitter apple peel	Carbonization	Crystal violet dye		(Aggarwal et al., 2020)
Carbon dots	Pear juice	Hydrothermal treatment	Methylene blue dye		(Das et al., 2019)
Nitrogen doped carbon dots	*Cocciniagrandis*	Hydrothermal treatment	Methyl orange dye		(Chandrasekaran et al., 2020)
Nitrogen doped carbon dots	*Citrus grandis*	Hydrothermal treatment	Methylene blue dye		(Ramar et al., 2018)
Nitrogen doped carbon dots	Empty fruit bunches	Hydrothermal treatment	Methylene blue and malachite green dyes		(Rani et al., 2021)
Nitrogen doped carbon dots	*Azadirachtaindica*	Hydrothermal treatment	Safranin-O dye		(Dhanush and Sethuraman, 2020)
Nitrogen doped carbon dots and chlorine doped carbon dots	Aqua mesophase pitch	Hydrothermal treatment	Rhodamine B, methylene blue and indigo carmine dyes		(Cheng et al., 2019)
Nitrogen and magnesium co-doped carbon dots	*Bougainvillea* leaves	Carbonization	Methylene blue dye		(Bhati et al., 2018)
Nitrogen and cobalt co-doped carbon dots	Vitamin B12	Pyrolysis	Rhodamine B dye		(Wang et al., 2020)
Sulfur and chlorine co-doped carbon dots	Palm powder	Hydrothermal treatment	Rhodamine B and methylene blue dyes		(Zhu et al., 2020)
Phosphorus doped carbon dots	D-glucose anhydrous	Hydrothermal treatment	Methylene blue dye		(Mathew et al., 2020)
Boron doped carbon dots	Citric acid	Hydrothermal treatment	Rhodamine B and methylene blue dyes	Photosensitizer Electron Mediator	(Peng et al., 2020)
Phenylhydrazine modified carbon dots	Maltitol	Thermal decomposition	Methylene blue dye		(Han et al., 2020)
TiO_2_-wsCQDs	Lemon peel	Hydrothermal treatment	Methylene blue dye		(Tyagi et al., 2016)
CQDs/TNTs	Citric acid	Hydrothermal treatment	Methylene blue dye		(Zhao et al., 2018)
CDs/N-TiO_2_	Ascorbic acid	Hydrothermal treatment	Rhodamine B dye		(Zhang J. et al., 2020)
Active blend functionalized TiO_2_	Sodium alginate	Microwave digestion	Methylene blue, crystal violet and methyl orange dyes and pharmaceuticals like diclofenac and tetracaine		(Vassalini et al., 2020)
N-CDs/TiO_2_	Citric acid	Hydrothermal treatment	Rhodamine B dye		(Ouyang et al., 2020)
NCQDs/TiO_2_	Citric acid	Microwave assisted method			(Li et al., 2018)
NP-CQDs/TiO_2_	Citric acid	Thermal treatment	Methylene blue dye		(Guo et al., 2018)
cl-Ch-p(VI)/TiO_2_NPs-CDs	Sugar cane juice	Microwave assisted method	Reactive Blue 4 and Reactive Red 15 dyes and toxic compound 2,4-dicholorophenol		(Midya et al., 2020)
CDs/P25/Rgo	Citric acid	Hydrothermal treatment	Rhodamine B, methylene blue and methyl orange dyes	Photosensitizer Electron Mediator Special Converter	(Zhu et al., 2021b)
CQDZ	Ammonium citrate	Thermal treatment	Methylene blue dye		(Ratnayake et al., 2019)
CDs_BZO	Citric acid	Hydrothermal treatment	Methylene blue dye		(Patra et al., 2018)
N,Fe-CDs/G-WO_3_	Folic acid	Hydrothermal treatment	Rhodamine B and methylene blue dyes and pharmaceuticals like ciprofloxacin, tetracycline hydrochloride and oxytetracycline		(Ni et al., 2020)
WO_3_/GO/NCQDs	Citric acid	Hydrothermal treatment	Methyl orange dye		(Jamila et al., 2020a)
ZnO/C-dots	Glucose and copra oil	Solvothermal treatment	Methylene blue dye		(Velumani et al., 2020)
ZnO-CDs	Grounded coffee	Hydrothermal treatment	Methylene blue dye		(Omer et al., 2018)
C_Dots_/ZnO_2_	D-glucose	Microwave assisted method	Methyl orange, methylene blue and Rhodamine B dyes		(El-Shamy, 2020b)
PVA/CZnO_2_	D-glucose	Microwave assisted method	Methylene blue dye		(El-Shamy, 2020a)
PVA/CQDs	D-glucose	Solution casting method	Methylene blue dye		(El-Shamy and Zayied, 2020)
PVP-CD	Lemon juice	Hydrothermal treatment	Rhodamine B, malachite green, crystal violet and Eosin Y dyes		(Nayak et al., 2020)
CDs/CeO_2_	Wood powder and citric acid	Hydrothermal treatment	Methylene blue dye		(Gong et al., 2019)
CuO/NCQDs	Citric acid	Hydrothermal treatment	Methyl orange dye	Photosensitizer Electron Mediator Special Converter	(Jamila et al., 2020b)
CQDs/KNbO_3_	L-ascorbic acid	Hydrothermal treatment	Crystal violet dye		(Qu et al., 2018)
Pd@CD-CONH	Citric acid	Hydrothermal treatment	Rhodamine B dye		(Selim et al., 2020)
MIL-53(Fe)/CQDs/MNPs	Wood based activated carbon	HNO_3_ treatment	Rhodamine B, malachite green and methylene blue dyes and Cr(VI)		(He et al., 2021)
CCN	Citric acid	Hydrothermal treatment	Methylene blue and Rhodamine B dyes		(Zhang L. et al., 2020)
NCQD/g-C_3_N_4_	Citric acid	Hydrothermal treatment	Methylene blue dye		(Seng et al., 2020)
GA-CQDs/CNN	EDTA-2Na•2H_2_O	Hydrothermal treatment	Methyl orange dye		(He et al., 2018a)
g-C_3_N_4_/Ag_3_PO_4_/NCDs	Citric acid	Solution process	Methylene blue and Rhodamine B dyes and refractory pollutant phenol		(Miao et al., 2018a)
Ag_3_PO_4_/GO/NCD	Citric acid	Hydrothermal treatment	Methylene blue and Rhodamine B dyes and refractory pollutant phenol		(Miao et al., 2018b)
CDC-dye	Citric acid	Microwave assisted method			(Jana et al., 2020)
N-CDs/m-TiO_2_	Citric acid		Methylene blue dye		(Zhang et al., 2021)
CDs/g-C_3_N_4_/SnO_4_	Quanidiniumhydrochoride	Thermal polymerization method	Indomethacin (IDM)		(Li et al., 2021)
ZnO/CQDs		Hydrothermal treatment	Benzene and Methanol		(Yu et al., 2012)
CDs/NiCO_2_O_4_		Hydrothermal treatment	Photocatalytic HER and OER		(Nie et al., 2021)
Au/CQDs			Cyclohexane		(Liu et al., 2014)
L-CQDs/ZnO		Hydrothermal treatment	Phenol		(Liang et al., 2020)
ZnO/CQDs/AuNPs	D-lactose	Hydrothermal treatment	Methylene blue dye		(Bozetine et al., 2021)
CQDs/Au/BMO	Citric acid	Hydrothermal treatment	Phenol		(Zhao et al., 2021)
CQDs/BiOCOOH/uCN	Citric acid	Hydrothermal treatment	Sulfathiazole (STZ)		(Hu et al., 2021)
N-CQDs/BiOI_x_Br_1-x_	Citric acid	Co-precipitation method	Phenol		(Gao et al., 2021)

## 2 Synthesis Methods of CQDs

**FIGURE 2:**
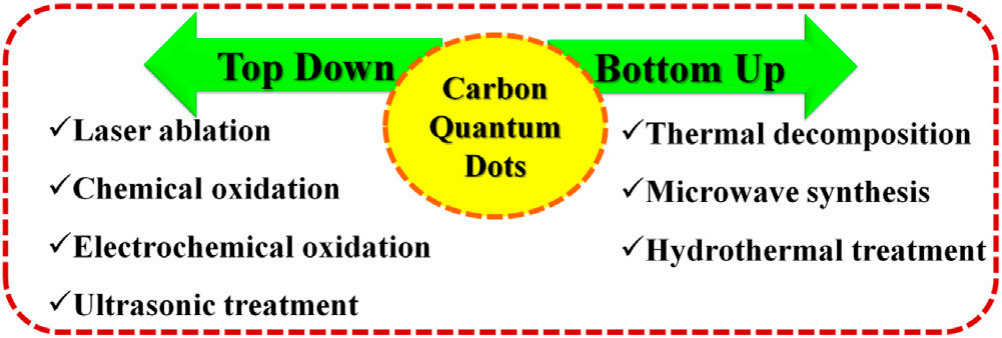
Schematic representation of CQDs synthesis by “top-down” and “bottom-up” approaches.

Xu et al. discovered fluorescent CQDs by coincidence while purifying single-walled carbon nanotubes (SWCNTs) from arc-discharged soot that they came up with the idea to publish their findings in Science (Xu et al., 2004). There have now been other alternative synthesis strategies for producing CQDs that have been found and refined. This research is primarily concerned with easy, cost-effective, size adjustable, and large-scale methodologies for synthesizing CQDs that have improved functions and can be produced in a wide range of compositions and structural arrangements. “Top-down” and “bottom-up” strategies are often used in the production of CQDs, and they may be separated from one another by the direction in which the size expansion of the implemented materials takes place: “top-down” and “bottom-up” procedures schematic representation in Figure 2.

Top-down techniques use treatments including arc discharge, laser ablation, electrochemical oxidation, chemical oxidation, and ultrasonic synthesis to create CQDs from macroscopic carbon structures such as graphite, activated carbon, and carbon nanotubes (Zhou et al., 2007; Yang et al., 2009a; Peng and Travas-Sejdic, 2009; Tian et al., 2009; Li X. et al., 2011; Tang D. et al., 2013; Hu et al., 2013b). Bottom-up procedures use microwave synthesis, thermal decomposition, hydrothermal treatment, template-based routes, and plasma treatment to make CQDs from molecular precursors such as citric acid, sucrose, and glucose (Zhu et al., 2009; Li et al., 2012; Tang et al., 2012; Chen B. et al., 2013; Qu et al., 2013; Bian et al., 2014; Ma et al., 2015).

### 2.1 Top-Down

#### 2.1.1 Laser Ablation

Recent trends in CQD preparation favor laser ablation, which can easily control morphology and manufacture a variety of nanostructures. Laser ablation is an excellent process for creating CQDs with a limited size distribution, good water solubility, and fluorescence properties, among other qualities. However, it is not widely used because of its intricate operation and high cost (Hu et al., 2009; Cui et al., 2020).

In two different configurations, a batch configuration and a flow jet configuration, Donate-Buendia et al. synthesized CQDs by laser irradiation of glassy carbon particles suspended in polyethylene glycol 200 (Doñate-Buendia et al., 2018).

#### 2.1.2 Chemical Oxidation

Chemical oxidation is a cost-effective and convenient method for large-scale production that does not require sophisticated devices. Tan et al. made CQDs *via* oxidation (Tan et al., 2019). In a 100 ml round-bottom flask, 50 ml of concentrated HNO_3,_ and HClO_4_ were combined 1:1 with 2.00 g coconut shell activated carbon. Oxidation was carried out for 120 min at 100°C with 500 rpm magnetic stirring (500 rpm). The dark suspension was cooled and then separated using a 1,000 Da ultrafiltration membrane in an MSC300 ultrafiltration apparatus. The filtrate was concentrated in vacuo and dialyzed for 3 days to remove inorganic ions to create CODs.

#### 2.1.3 Electrochemical Oxidation

In the production of CQDs, electrochemical oxidation is the most often used technique. This approach has the benefits of high purity, cheap cost, high yield, ease of size modification, and excellent repeatability (Zhou et al., 2021). Zhou et al. reported the first electrochemical generation of CQDs from multiwalled carbon nanotubes (MWCNTs) (Zhou et al., 2007). Li et al. described the direct electrochemical production of 3–5 nm green luminous GQDs (Li Y. et al., 2011).

#### 2.1.3 Ultrasonic Synthesis

Although it is widely established that ultrasound can generate alternate low-pressure and high-pressure waves in liquids, the development and collapse of small vacuum bubbles are not well understood (Liang et al., 2013; Lu and Zhou, 2019; Wu et al., 2019; Rosiles-Perez et al., 2020).

Qi et al. synthesized CQDs by ultrasonic synthesis 60 ml l-glutamic acid aqueous (0.45 M) introduced to the reaction kettle through an ultrasonic transducer. The ultrasonic generator remained at 50% power during the reaction. The solution was heated to a particular temperature and then cooled to room temperature when it became yellow and no visible precipitation occurred, indicating the CQDs were formed (Qi et al., 2021).

### 2.2 Bottom-Up

#### 2.2.1 Thermal Decomposition

The thermal breakdown has been utilized in the past to produce various semiconductor and magnetic nanomaterials, among other things. Recent research has demonstrated that external heat can contribute to the dehydration and carbonization of organic materials, resulting in the formation of CQDs. The advantages of this process include ease of operation, a solvent-free approach, a wide range of precursor tolerance, a fast reaction time, a low cost, and the ability to scale up production (Chen et al., 2018; Ghosh et al., 2021; Kang et al., 2022; Ðorđević et al., 2022).

Tang et al. created R-CQDs by heat treatment wine lees, which served as the carbon source (Tang et al., 2021). By an open beaker, 100 ml of wine lees was heated for 0.5–2 h on a heating platform at 300°C to get the reactants, and then 100 ml of ethanol solution was poured into the beaker once the reactants had cooled. For regulating CQD size and homogeneity, the supernatant was post-treated using column chromatography or dialysis followed with evaporation of ethanol yields CQDs.

#### 2.2.2 Microwave Synthesis

Microwaves are a form of electromagnetic wave with a broad wavelength range of 1 mm to 1 m, and they can deliver massive amounts of energy to a substrate, allowing it to be broken apart chemically. Therefore, the microwave approach may be used to significantly reduce reaction time while also providing homogenous heating, which results in a more consistent distribution of quantum dots in the final product (Yang et al., 2018; Zhu J. et al., 2021).

Yu et al. prepared CQDs by a microwave synthesis method (Yu et al., 2018). In a 100 ml beaker, phthalic acid (2 g) and triethylenediamine hexahydrate (1 g) were dissolved in 3 ml deionized water. The beaker was then cooked for 60 s on the revolving plate of a household MW oven (700 W). After cooling, the crude products were dialyzed for 24 h against 500 ml deionized water to make powdered CQDs. Basoglu et al. synthesized CQDs by the microwave-assisted pyrolysis of the roasted chickpeas (Başoğlu et al., 2020). In total, 2 g of roasted chickpeas were digested in 40 ml ultra-pure water. This mixture was transferred to a 250 ml beaker and microwaved for 2 min. 15 min at 3,000 rpm centrifuged the cooled solution. The Allegra X-30R was utilized (Beckman Coulter). The cream solid was extracted from the supernatant. The liquid was filtered using 0.45 and 0.2 mm syringe membranes. The solution was centrifuged for 15 min at 12,000 rpm to remove the aggregates. The liquid CQD was blended. Solute was chilled at 4°C to make CQDs.

#### 2.2.3 Hydrothermal Treatment

Hydrothermal carbonization produces innovative carbon-based compounds from saccharides, organic acids, juice, or discarded peels. Generally, an organic precursor solution is enclosed and heated in a hydrothermal reactor.

Das et al. fabricated green-emissive carbon quantum dots (CQDs) from pear juice in a simple and scalable hydrothermal route (Das et al., 2019). In order to make the CQDs, the pear juice was held in a Teflon-lined autoclave at 180°C for 36 h and filtered with a 0.22 µm filter. Chandrasekaran et al. prepared nitrogen-doped carbon dots (N-CDs) from *Coccinia grandis* extract by a simple hydrothermal method (Chandrasekaran et al., 2020). The *Coccinia grandis* extract and aqueous ammonia were held in a Teflon-lined autoclave for 12 h at 180°C, filtered with Whatman 40 filter paper, and centrifuged at 1,000 rpm for 1 h to make N-CDs.

## 3 Properties of CQDs

### 3.1 Optical Absorbance

For the most part, the optical absorption peaks of CQDs in the UV visible region are interpreted as being caused by the sp^2^ conjugated carbon p-p transition and the n-p transition caused by the hybridization with heteroatoms such as N, S, and P, among others. Surface passivation or modification processes can be used to control the absorption property of the surface. He et al. synthesized CODs from lemon juice using a simple hydrothermal treatment at low temperatures and short time (He M. et al., 2018). The CQDs have good optical and material qualities. Under UV or blue light irradiation, they produce strong blue-green fluorescent light. According to He et al., CQDs can image plant cells. These critical insights can help us learn more about CQDs and investigate their potential applications.

### 3.2 Fluorescence

PL is one of the most intriguing characteristics of CQDs, both from a basic and an application-oriented standpoint. Section 4 describes how to modify the PL features of CQDs to get the desired result. Raj et al. (2021) synthesized water-dispersible and fluorescence-stable carbon quantum dots (CQDs) at a gram scale. Their optical and fluorescent properties were studied in depth. Aquatic dispersion emits intense yellow light in UV lamps (365 nm). As a nano-probe CQDs can detect heavy metal ions like Cr^3+^, Fe^3+^, and Cu^2+^ in aqueous media at neutral pH by quenching their fluorescence. The LOD of 100 nM for each of these ions of CQDs. These fluorescence-stable CQDs are easily manufactured and maintained for sensing applications.

### 3.3 Phosphorescence

CQDs have recently been discovered to have phosphorescence capabilities (Tan et al., 2016a, Tan et al., 2016b; Chen et al., 2019; Yuan et al., 2022). It has been possible to develop a pure organic room temperature phosphorescent (RTP) material based on water-soluble CQDs, and the phosphorescent lifetime of this material has been increased to the sub-second order (380 ms). It was possible to witness clear phosphorescence at room temperature when the CQDs were dispersed in a polyvinyl alcohol (PVA) matrix when the PVA matrix was activated with UV light (Deng et al., 2013). Preliminary research revealed that the phosphorescence was caused by triplet excited states of aromatic carbonyls on the surface of the CQDs, which was supported by the results of the experiments. The hydrogen bonding between the matrixes PVA molecules can successfully preserve the triplet excited state energy from rotational or vibrational loss by rigidifying the groups that make up the triplet excited state. Section 4 discussed more related literature on CODs.

### 3.4 Chemiluminescence

The chemiluminescence (CL) of CQDs was initially observed when they were mixed with oxidants such as potassium permanganate (KMnO_4_) and Cerium (IV) (Lin et al., 2012). EPR shows that oxidants such as KMnO4 and Cerium (IV) may insert holes into CQDs. This increases the population of holes in CQDs and speeds up electron-hole annihilation, leading to CL emission. Moreover, the CL intensity was dependent on CQD concentration. The thermal equilibrium of electron dispersion in CQDs also discovered that increasing temperature had a beneficial influence on CL. The fact that the surface groups of this system’s CL characteristics can be changed is intriguing (Teng et al., 2014). The CL of CQDs offers new possibilities for their use in reductive substance determination (Lin et al., 2011; Zhao et al., 2013; Wang et al., 2019; Shen et al., 2020).

### 3.5 Photoluminescence

There has been a significant increase in recent years in the amount of research being done on the PL of CQDs, which is one of the most exciting characteristics of CQDs and has been used in the field of photocatalysis (Chen et al., 2022; Peng et al., 2022). The PL emission pattern is similar to the Stokes type emission pattern in that the PL emission wavelength is longer than the excitation wavelength of the laser. Many publications have been published on the observation of PL emissions in CQDs from diverse sources (Cao et al., 2022; Liang et al., 2022; Pooresmaeil et al., 2022; Yu et al., 2022; Zhang et al., 2022). A detailed look at the spectroscopic aspects of the emissions and the underlying structural characteristics reveals that most recorded PL emissions fall into one of two groups. One is related to band gap transitions corresponding to conjugated p-domains, while the other is due to defects in graphene structures. The two groups are often interrelated since the exploitation or manipulation of graphene sheet defects creates or inducts p-domains. Many investigations examined the relationship between PL emission and CQD excitation wavelength discussed in Section 4.

## 4 Photocatalytic Applications of CQDs

There are some advantages to using CQDs in photocatalysis. CQDs are superior to other typical photocatalysts in terms of water solubility, chemical stability, and low toxicity (e.g., ZnO, TiO2, and CdS). After surface modification, CQDs display outstanding and tunable optical characteristics of absorbance and PL. UCPL of CQDs, in particular, may greatly increase the sunlight absorption of wide band gap semiconductors into the visible and near-infrared regions. Photoinduced CQDs are also good electron donors and acceptors, allowing for efficient electron-hole separation. Thus, CQDs can be used as electron mediators, photosensitizers, spectral converters, and sole photocatalysts. In fact, these many impacts often occur concurrently. CQD-based photocatalyst systems are summarized in this section.

### 4.1 Carbon Quantum Dots (CQDs)

Roushani et al. fabricated graphene quantum dots (GQDs) by pyrolyzing citric acid (CA) as a source of carbon (Roushani et al., 2015). The CA was held in a beaker on a heating mantle at 200°C for 30 min and then neutralized with NaOH to make GQDs. According to the photoluminescence (PL) spectrum, the GQDs showed an emission peak at 460 nm with an excitation wavelength of 362 nm, indicating that GQDs are fluorescent in nature. The TEM result demonstrated that the GQDs had an average diameter of 15 nm. The zeta potential of the GQDs was −24.6 mV, indicating that the GQDs had a negatively charged surface. The Raman spectrum confirmed the presence of G-band and D-band at 1,600 and 1,377 cm^−1^, respectively. The I_D_/I_G_ value of GQDs was as high as 1.03, indicating GQDs have a crystal structure with some sp^2^ defects caused by smaller clusters. The GQDs were used as photocatalysts for the degradation of New Fuchsin (NF) dye under visible light. The effects of GQDs, initial NF concentration, pH of dye, and contacting time on the degradation efficiency were investigated. The rate constant and degradation efficiency were rarely affected by NF concentration and only decreased slightly when the initial NF concentration increased.

Zaib et al. synthesized carbon dots (CDs) from *Elettaria cardamomum* in an eco-friendly way by the facile sonication method (Zaib et al., 2021). The *Elettaria cardamomum* leaves were ultra-sonicated for 45 min, centrifuged at 4,500 rpm for 15 min, and filtered with 0.22 μm membrane filter to make CDs. In the XRD result, the peak at 22.9° confirmed the existence of amorphous phase carbon in the synthesized CDs. The Raman spectroscopic result showed a G-band at 1,575 cm^−1^, which represents a graphitic band of carbon, and a weaker D-band at 1,365 cm^−1^, which represents a disordered band of carbon. According to the photoluminescence spectroscopic result, when the excitation wavelength was 514 nm, CDs showed two emission peaks at 520 and 850 nm, respectively, indicating that CDs are fluorescent. The CDs were used as photocatalysts for the degradation of methylene blue (MB) and congo red (CR) under visible light irradiation. At the optimal condition (4 pH, 5 ppm CR, and 5 ml CDs), CR degradation took 50 min. However, when the dye was changed to MB with a similar concentration and at 8 pH, degradation took just 5 min more than CR.

Aggarwal et al. prepared photoactive carbon dots (CDs) through a facile, green, and scalable method by charring bitter apple peel (Aggarwal et al., 2020). Dried peel was carbonized at around 300°C in a muffle furnace for 2 h to generate carbon dots. XPS study showed the elemental composition of CDstobe 60.3, 37.47, and 2.23% for C, N, and O, respectively. The presence of graphitic G-band and disordered D-band at 1,571 and 1,355 cm^−1^ was confirmed by Raman spectroscopy. H-TEM studies showed the presence of graphitic fringes in CDs. The photoactive CDs were used as photocatalysts for the degradation of crystal violet (CV) under solar light. The photoactive CDs degraded 20 ppm of CV in ∼90 min, much faster than the degradation in the dark. According to the active species scavenging experiment, the most important active species engaged in the degradation reactions were photogenerated electrons and holes. The degradation of CV, from aromatic compound to little aliphatic pieces, was confirmed by NMR spectroscopy.

**FIGURE 3:**
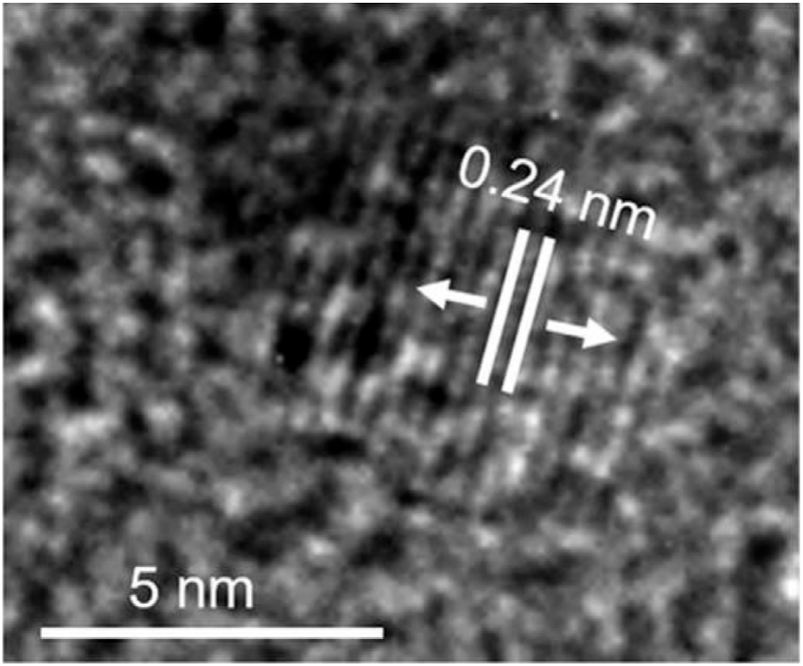
High resolution transmission electron microscopic (H-TEM) image of a single CQD showing graphitic spacing. (Reproduced from Das et al., 2019, Springer Nature).

**SCHEME 1:**
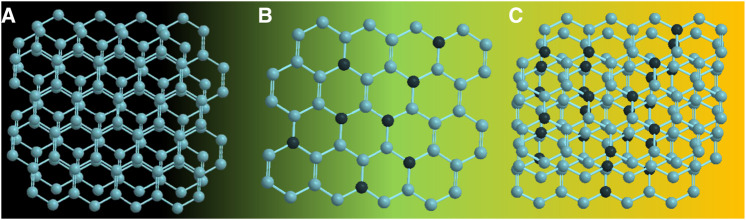
Schematics of **(A)** unmodified, **(B)** modified, and **(C)** composite carbon quantum dots (CQDs).

Das et al. fabricated green-emissive carbon quantum dots (CQDs) from pear juice in a simple and scalable hydrothermal route (Das et al., 2019) and H-TEM analysis shows the high crystallinity of CQDs in Figure 3. According to XPS results, the elemental composition of CQDs was 71% and 29% for C and O, respectively. The photoluminescence (PL) results revealed that the CQDs showed excitation-dependent emission. As the excitation wavelength increased from 380 to 600 nm, emission wavelength increased from 470 to 650 nm with gradually decreasing emission intensity. The CQDs were used as photocatalysts for the degradation of methylene blue (MB) under visible light. 99.5% of MB was degraded within 130 min. The excellent photocatalytic performance was due to efficient transfer and separation of photogenerated charge and good light-harvesting capability. Besides, the CQDs were used as selective sensors for Fe (III) and ascorbic acid (AA). The CQDs stopped emitting fluorescence when they were bound to Fe (III) and began to emit fluorescence again when AA was added. Accordingly, Scheme 1 demonstrates the various types of CQDs Scheme 1A Unmodified, Scheme 1B Modified and Scheme 1C Composite Carbon Quantum Dots (CQDs).

### 4.2 Modified Carbon Quantum Dots (CQDs)

Chandrasekaran et al. prepared nitrogen-doped carbon dots (N-CDs) from *Coccinia grandis* extract by a simple hydrothermal method (Chandrasekaran et al., 2020). According to H-TEM results, N-CDs had spherical shapes with a diameter of 3.3 nm. The EDS result demonstrated that N-CDs had a composition of C (91.55%), N (2.12%), and O (5.48%). In XRD results, the peak at 28.5° confirmed the existence of amorphous carbon in N-CDs. N-CDs were used as catalysts for the reduction of methyl orange (MO) with NaBH_4_. The MO reduction rate constant was 0.1263 min^−1^, which was higher than that of using pure NaBH_4_.

Ramar et al. (2018) synthesized undoped carbon quantum dots (UCQDs) and N-doped carbon quantum dots (NCQDs) using Pomelo juice *via* a simple and eco-friendly hydrothermal process. The Pomelo juice was held in a Teflon-lined autoclave at 200°C for 7 h and centrifuged for 30 min at 12,000 rpm to make UCQDs. The Pomelo juice and ammonium bicarbonate were held in a Teflon-lined autoclave at 200°C for 7 h and centrifuged for 30 min at 12,000 rpm to make NCQDs. In the XRD results, the peak at 22.4° and the peaks at 12.6° and 28.2° confirmed the presence of amorphous carbon in UCQDs and NCQDs, respectively. According to TEM results, UCQDs and NCQDs had average diameters of 3 and 70 nm, respectively. The Raman spectroscopy demonstrated that the D-band, corresponding to disordered carbon, was shifted from 1,354 to 1,335 cm^−1^, and the G-band, corresponding to graphitic carbon, was shifted from 1,571 to 1,563 cm^−1^ when CQDs were N-doped. Both CQDs acted as good photocatalysts for the degradation of methylene blue (MB) under solar light, which followed pseudo-first-order kinetics. CQDs showed brilliant photocatalytic performance because they have high transfer efficiency of photoinduced electrons and remarkable sunlight absorption abilities.

Rani et al. (2021) synthesized N-doped carbon quantum dots (NCQDs) from empty fruit bunches (EFB) by a green hydrothermal method. The lignin extracted from EFB, urea, and deionized water was held in a Teflon-lined autoclave for 8 h at 180°C and filtered with 20–25 μm filter paper to make NCQDs. The EDX result showed that the elemental composition of NCQDs was 60.76, 9.01, 20.38, 5.92, and 3.93% for C, N, O, Na, and Cl, respectively. According to the TEM analysis, the NCQDs had diameters of 3.6 nm. The zeta potential of NCQDs was −20.35 ± 0.52 mV, indicating that the NCQDs had a negatively charged surface. With NCQDs, 97% of methylene blue (MB) and 98% of malachite green (MG) were degraded in 180 and 120 min under solar light. Without NCQDs, only 59% of MB and MG were degraded. The excellent photocatalytic activity of NCQDs was because of the separation of photogenerated charge and suppressed electron-hole recombination. The photocatalytic activity of NCQDs was superior to that of CQDs owing to the good fluorescent properties gained from N-doping. The NCQDs could be used at least ten times without significant performance decrease due to their high stability.

Dhanush et al. prepared fluorescent phyto-derived nitrogen-doped carbon dots (PDNCDs) from neem seeds using a green hydrothermal method (Dhanush and Sethuraman, 2020). The neem seeds and aqueous ammonia were held in an autoclave with Teflon lining for 12 h at 180°C, filtered with Whatman 40 filter paper, and centrifuged to make PDNCDs. According to H-TEM results, PDNCDs had diameters of 2.5 nm. In the XRD result, the peak at 28.47° confirmed the existence of amorphous carbon in PDNCDs. The Raman spectroscopic result showed a G-band at 1,606 cm^−1^, which represents a disordered band of carbon, and a weaker D-band at 1,369 cm^−1^, which represents a graphitic band of carbon. The properties of PDNCDs could be precisely tuned using different solvents. PDNCDs were used as catalysts for the reduction of Safranin-O dye with NaBH_4_. Safranin-O was reduced in 6 min, which was better than that of pure NaBH_4_ and undoped CDs. The catalytic activity of PDNCDs came from their ability to transfer electrons from NaBH_4_ to Safranin-O.

Cheng et al. synthesized fluorescent nitrogen-doped carbon quantum dots (N-CQDs) and chlorine-doped carbon quantum dots (Cl-CQDs) from aqua mesophase pitch (AMP) through a hydrothermal process (Cheng et al., 2019). The AMP solution was held in a polytetrafluoroethylene-lined autoclave at 120, 150, and 180°C for 12, 24, and 48 h, respectively, and centrifuged for 10 min at 8,000 rpm to make CQDs. Then, the CQDs held at 120°C for 24 h (CQDs-120-24) and ammonia were kept for 12 h at 120°C in the autoclave and kept for 0.5 h at 80°C in an open space to make N-CQDs. CQDs-120-24 and thionyl chloride went through the same procedure to make Cl-CQDs. The CQDs, N-CQDs, and Cl-CQDs had diameters of 2.8, 4.5, and 4.2 nm, respectively, according to the TEM results. Furthermore, the PL results demonstrate that CQDs had a quantum yield (QY) of 27.6%, while N-CQDs and Cl-CQDs had lower quantum yields. In the H-TEM results of CQDs, N-CQDs, and Cl-CQDs, interlayer spacing was 0.33 nm, corresponding to the (002) spacing of graphitic carbon. XPS results demonstrated that the CQDs contained C and O; N-CQDs contained C, N, and O; and Cl-CQDs contained C, Cl, and O. The CQDs, N-CQDs, and Cl-CQDs were used as photocatalysts for the degradation of rhodamine-B (Rh B), methylene blue (MB), and indigo carmine (IC) under sunlight. The N-CQDs exhibited the highest Rh B degradation efficiency of 97% in 4 h and a rate constant of 0.02463 min^−1^. The degradation efficiency was maintained at 93% during five degradation cycles. The Cl-CQDs displayed the highest MB and IC degradation efficiency of 56% and 60%, respectively. The photocatalytic performances of N-CQDs and Cl-CQDs were better than that of pristine CQDs because the electric field was generated in N-CQDs and Cl-CQDs.

**FIGURE 4:**
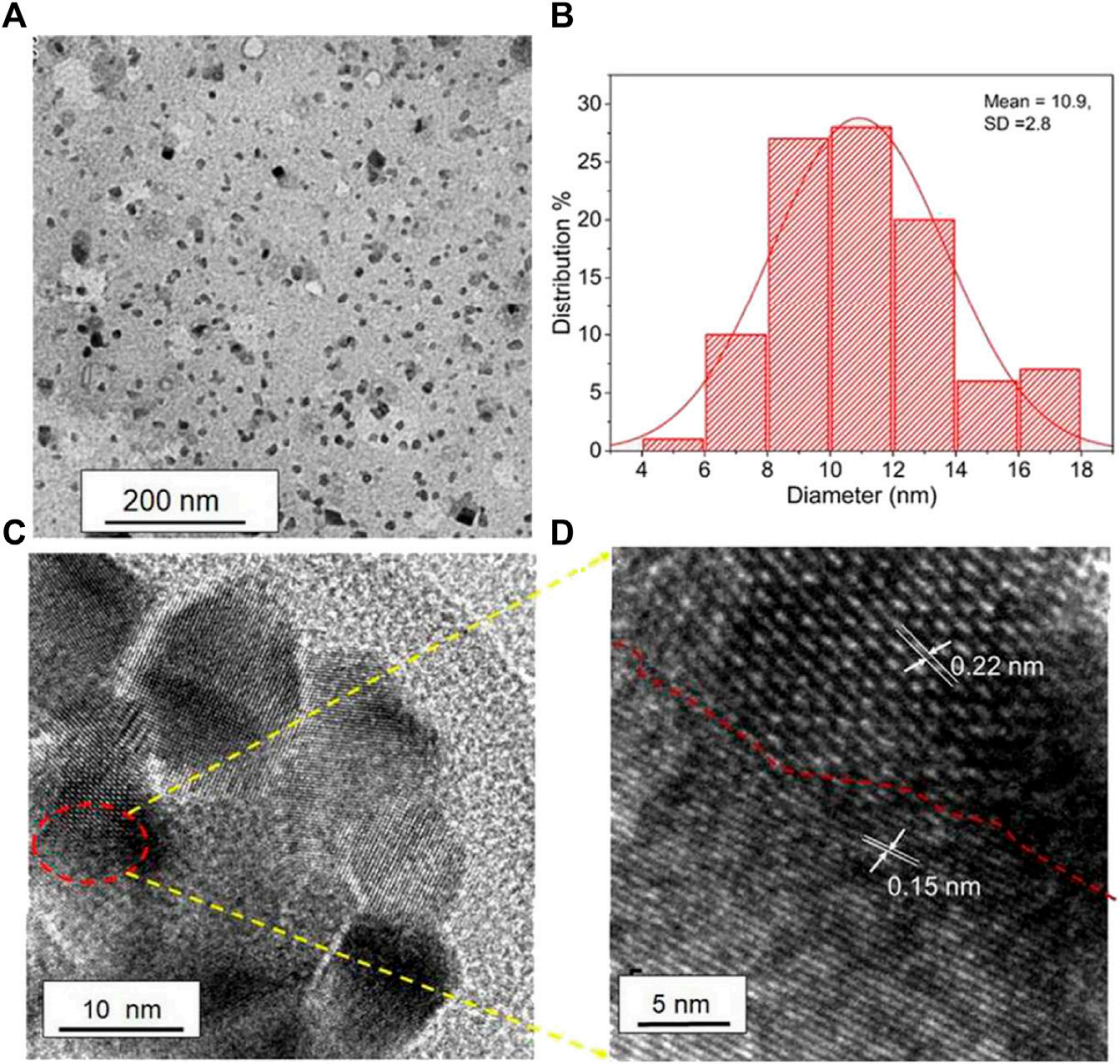
Different magnification **(A)** TEM image of r-Mg-N-CD and **(B)** its corresponding size distribution; **(C)** HRTEM image of r-Mg-N-CD and **(D)** zoomed image of **(C)**. (Reproduced with permission from Bhati et al., 2018, Copyright © 2018, American Chemical Society).

Bhati et al. prepared red-emitting-magnesium-nitrogen-embedded carbon dots (r-Mg-N-CD) in an eco-friendly way (Bhati et al., 2018). Bougainvillea leaves extract was carbonized in a 1400 W microwave oven for 15 min, sonicated, and centrifuged for 30 min at 7,000 rpm to make r-Mg-N-CD. The H-TEM results showed in (Figure 4) the graphitic fringes of r-Mg-N-CD. According to the photoluminescence (PL) results, the r-Mg-N-CD showed excitation-independent emissions at ∼678 nm, a high quantum yield of ∼40%, and high photostability. According to XPS results, the elemental composition of r-Mg-N-CD was 76.7, 11.4, 6.9, and 5.0% for C, O, N, and Mg, respectively. The r-Mg-N-CD was used as a cheap photocatalyst for aqueous phase photodegradation of a pollutant dye, methylene blue (MB), under sunlight. The photodegradation under sunlight was about six times faster than that under visible light from the artificial tungsten bulb, which only took ∼120 min.

Wang et al. developed single cobalt atoms anchored carbon dots (CoSAS@CD) (Wang et al., 2020). Vitamin B_12_ was held in a quartz boat for 2 h at 250°C; was hydrolyzed with NaOH for 24 h at 80°C; and went through filtration, dialysis for 3 days, and freeze-drying to make CoSAS@CD. The TEM results showed that CoSAS@CD were spheres with the size of 9.0 nm. According to XRD results, the broad peak at 22° confirmed that CoSAS@CD was slightly graphitic. XPS results revealed that the elemental composition of CoSAS@CD was 64.73, 9.05%, 22.03%, and 4.19% for C, N, O, and Co, respectively. The photoluminescence (PL) results demonstrated that CoSAS@CD exhibited the strongest emission at 415 nm when the excitation wavelength was 300 nm. The CoSAS@CD was used as a photocatalyst for thee oxidation reactions under visible light irradiation. The CoSAS@CD exhibited high oxygen evolution rate of 168 μmol/h/g during the water oxidation reaction, high conversion of ∼90%, and high selectivity over 99% in imine formation reaction and oxidation degradation time of 10 min against RhB. The excellent photocatalytic activity of the CoSAS@CD came from improved visible light-harvesting ability and enhanced photoinduced charge transfer and separation.

Zhu et al. fabricated S/Cl co-doped biomass-based CDs (Bio-CDs) using palm powders through the hydrothermal process (Zhu et al., 2020). Palm powders and thionyl chloride were held in a Teflon-lined autoclave for 7 h at 200°C, filtered with a 0.22 μm syringe filter, and went through dialysis for 2 days, with freeze-drying to make Bio-CDs. According to the HTEM results, the Bio-CDs were spherical, had diameters of 3.54 nm, and displayed graphitic fringes. XPS result showed that the elemental composition of Bio-CDs was 42.20%, 43.36%, 10.02%, and 4.3% for C, O, S, and Cl, respectively. According to photoluminescence (PL) spectroscopy results, Bio-CDs showed excitation-dependent blue fluorescence with the strongest emission at 425 nm when the excitation wavelength was 340 nm. The Bio-CDs were used as photocatalysts for the degradation of rhodamine B (RhB) and methylene blue (MB) under visible light irradiation. The Bio-CDs displayed high RhB and MB degradation efficiency of ∼71.7% and ∼94.2%, respectively. The excellent photocatalytic activity was due to S/Cl co-doping and quantum confinement effect.

Mathew et al. fabricated carbon nano-dots (CD) and P-doped carbon nano-dots (P-CD) from d-glucose anhydrous *via* hydrothermal treatment (Mathew et al., 2020). Glucose was held in an autoclave with Teflon lining for 6 h at 150°C, filtered, and centrifuged at 5,000 rpm to make CD. Glucose and orthophosphoric acid were held in a Teflon-lined autoclave for 6 h at 110°C, neutralized with NaOH, filtered, and centrifuged at 5,000 rpm to make P-CD. The HTEM image showed that P-CD was quasi-spherical and had a radius of 2.19 nm. According to XPS result, the elemental composition of P-CD was 37%, 51.43%, 4.49%, and 7.08% for C, O, P, and Na, respectively. The photoluminescence (PL) spectra showed that P-CD exhibited the strongest emission at 524 nm at the excitation wavelength of 420 nm. The P-CD and CD were used as photocatalysts for the degradation of methylene blue (MB) under solar light. The P-CD and CD exhibited degradation rate constants of 0.02736 and 0.01645 min^−1^, which were much higher than the self-degradation rate constant of MB. The P-CD degraded about 85% of MB in 180 min. The enhanced photocatalytic activity of P-CD was due to the large surface area coming from the small size of P-CD.

Peng et al. synthesized “B-doped” carbon dots (C-dots) through a hydrothermal treatment (Peng et al., 2020). Citric acid, 1, 2-diboranyethane, and anhydrous dimethylformamide were held in a Teflon-lined autoclave for 6 h at 160°C, centrifuged at 3,000 rpm for 15 min, and subjected to dialysis for 3 days to make “B-doped” C-dots. In the TEM result, the absence of noticeable lattices showed that the “B-doped” C-dots were amorphous, not graphitic. The zeta potential of “B-doped” C-dots was −11.6 mV, indicating that the “B-doped” C-dots were negatively charged. According to photoluminescence (PL) results, the “B-doped” C-dots exhibited excitation-dependent emission, with the strongest emission at 450 nm when the excitation wavelength was 360 nm. The XPS results confirmed that the elemental composition of “B-doped” C-dots was 75.8%, 19.1%, and 5.1% for C, O, and N, respectively. The “B-doped” C-dots were used as photocatalysts for the degradation of rhodamine B (RhB) and methylene blue (MB) under visible light irradiation. RhB and MB were completely degraded in 170 min. The rate constants for RhB and MB degradation were 1.8 × 10^−2^ and 2.4 × 10^−2^ min^−1^. The “B-doped” C-dots showed good photocatalytic activity, which was better than other C-dots photocatalysts and comparable to metal-containing photocatalysts.

Han et al. prepared carbon quantum dots (CQDs) by a simple and scalable method (Han et al., 2020). Maltitol and hydrogen peroxide were held in a glass petri dish for 20 min at 200°C and centrifuged for 20 min at 13,000 rpm to make CQDs. Phenylhydrazine (PH), HCl, CHCl_3_, and CQDs were stirred for 36 h and filtered. Then, the precipitate was washed with CHCl_3_ and dried at 40°C in a vacuum for 24 h to make CQDs modified with PH (CQDs-PH). XPS result revealed that the elemental composition of CQDs was 57.6% and 42.4% for C and O, respectively. Raman spectroscopy confirmed the presence of a D-band at 1,354 cm^−1^ and a G-band at 1,595 cm^−1^, corresponding to disordered graphitic carbons, respectively. HTEM result demonstrated the presence of lattice fringes of graphene in CQDs. According to photoluminescence (PL) spectroscopy results, CQDs exhibited the strongest emission at 452 nm when the excitation wavelength was 360 nm. The CQDs/peroxymonosulfate (PMS) system was used in the photo-degradation of dyes under visible light irradiation and showed good photocatalytic activity and high stability. The alkaline condition was preferred in dye degradation because the alkaline condition promoted the separation of charge. CQDs modified with phenylhydrazine (CQDs-PH) displayed the strongest photocatalytic PMS activation for methylene blue (MB) degradation, showing a degradation time of ∼5 min. By radical quenching tests, the MB degradation mechanism was revealed. Photogenerated electrons were reacted with PMS to produce O_2_^∙−^. Then, O_2_^∙−^ and photogenerated h^+^ degraded MB.

### 4.3 Composite Carbon Dots

Tyagi et al. prepared water-soluble carbon quantum dots (wsCQDs) from lemon peel *via* a simple and cheap hydrothermal method (Tyagi et al., 2016). The lemon peel was held in a Teflon-lined autoclave for 12 h at 200°C, washed with dichloromethane, centrifuged for 30 min at 10,000 rpm, and dried at 100°C to make wsCQDs. According to TEM images, the wsCQDs were spherical and had diameters of 1–3 nm. XPS result demonstrated that the wsCQDs contained C and O. The photoluminescence (PL) spectra showed that the wsCQDs had photoluminescent properties with a quantum yield of ∼14%. As excitation wavelength increased from 300 to 540 nm, emission wavelength also increased. The emission intensity was highest when the excitation wavelength was 360 nm. The wsCQDs were used as sensitive and selective fluorescent probes for Cr^6+^ ions and showed a detection limit of ∼73 nM. Polyvinyl pyrrolidone solution and titanium isopropoxide solution were mixed, made into nanofibers with a syringe, and calcined in a muffle furnace for 2 h at 500°C to make TiO_2_ nanofibers. TiO_2_ nanofibers were functionalized with 6-aminohexanoic acid and were mixed with wsCQDs solution to make TiO_2_-wsCQDs nanocomposite. TiO_2_-wsCQDs nanocomposite was used as a photocatalyst for the degradation of methylene blue (MB) under visible light. The TiO_2_-wsCQDs nanocomposite displayed degradation efficiency that was about 2.5 times higher than that of TiO_2_ nanofibers. The photocatalytic performance was enhanced because wsCQDs promoted charge separation.

Zhao et al. developed a carbon quantum dots/TiO_2_ nanotubes (CQDs/TNTs) nanocomposite by an enhanced hydrothermal method by anchoring CQDs onto the outer surface of TNTs (Zhao et al., 2018). TiO_2_ nanoparticles and NaOH were held in a Teflon-lined autoclave for 36 h at 135°C, treated with HCl, stirred overnight, neutralized with DI water, and dried for 24 h at 80°C to make TNTs. Citric acid and ethylene diamine were held in the autoclave for 5 h at 150°C and subjected to dialysis for 2 days to make CQDs. TNTs were dispersed in CQDs solution and stirred for 24 h, centrifuged for 30 min at 10,000 rpm, and dried for 12 h at 70°C to make CQDs/TNTs. HTEM image showed (101) plane of TiO_2_ and (100) plane of CQDs. Energy dispersive spectroscopy (EDS) revealed that the elements weight of CQDs/TNTs was 20.3%, 63.31%, and 16.39% for Ti, O, and C, respectively. XRD pattern of CQDs/TNTs exhibited characteristic peaks of TiO_2_ at 25.1°, 28.2°, 39.39°, and 48.42°. The CQDs/TNTs nanocomposite was used as a photocatalyst for the degradation of 30 mg/L methylene blue (MB) under visible light irradiation. The CQDs/TNTs-0.2 (0.2 g of TNTs) showed the highest MB degradation efficiency of 91.3% in 50 min, which was two times higher than that of pristine TNTs. The photocatalytic performance was improved because the up-conversion photoluminescence (UCPL) properties of CQDs enabled more efficient use of visible light. Furthermore, the photoinduced electrons of TNTs were transferred to CQDs, and the electron-hole recombination was delayed. The π-π stacking between CQDs and dyes enhanced the adsorption ability of the CQDs/TNTs nanocomposite.

Zhang et al. constructed a carbon quantum dots/nitrogen doping TiO_2_ (CDs/N-TiO_2_) nanocomposite (Zhang J. et al., 2020). Ascorbic acid and ethanol were held in a high-pressure reactor for 3 h at 160°C to make CDs. The mixture of tetrabutyl titanate, anhydrous ethanol, nitric acid, and urea was held in a high-pressure reactor at 240°C for 10 h, dried at 110°C, and calcined at 200°C for 6 h to make N-TiO_2_. N-TiO_2_ and CDs were stirred for 1 h, centrifuged, washed with distilled water, and dried overnight at 90°C to make CDs/N-TiO_2_. The XRD pattern of CDs/N-TiO_2_ showed peaks at 25.3°, 37.8°, 48.0°, 53.9°,55.1°, 62.7°, and 75.0°, representing the (101), (004), (200), (105), (211), (204), and (215) planes of anatase phase. HTEM images confirmed the (101) plane of the anatase and (100) plane of the graphene carbon. According to XPS results, the CDs/N-TiO_2_ contained C, O, N, and Ti. Photoluminescence (PL) spectra revealed that 30CDs/N-TiO_2_ exhibited the lowest PL intensity, indicating the lowest recombination rate of charge carriers, which are used for photocatalytic degradation. The CDs/N-TiO_2_ nanocomposite was used as a photocatalyst for the degradation of 10 mg/L rhodamine B (RhB) under visible light and the reduction of 20 mg/L Cr (Ⅵ) under sunlight. The 30CDs/N-TiO_2_ (30 μL of CDs) nanocomposite displayed RhB degradation efficiency of 99.8% in 5 min, which was higher than that of TiO_2_ (6.66%) and N-TiO_2_ (19.97%). The 30CDs/N-TiO_2_ nanocomposite exhibited kinetic constants of RhB degradation 85.52 and 25.40 times higher than those of TiO_2_ and N-TiO_2_. With the 30CDs/N-TiO_2_ nanocomposite, Cr (Ⅵ) reduction took only 4 min. The photocatalytic activity was enhanced because N doping shifted absorption range to visible light range and made TiO_2_ behave like a p-type semiconductor to transfer photogenerated electrons to CDs more easily. Moreover, CDs stored received electrons to promote the photo-induced charge separation.

Vassalini et al. synthesized an active blend consisting of carbon-based nanoparticles, alginate, and organic acids in an eco-friendly way (Vassalini et al., 2020). Sodium alginate solution was sonicated for 15 min and held in a digester at 200°C for 15 min to make the active blend. The photoluminescence (PL) spectrum showed that the active blend emitted blue PL when the excitation wavelength was 365 nm. Raman spectroscopy confirmed the presence of D-band at 1,370 cm^−1^, corresponding to disordered carbons, and G-band at 1,550 cm^−1^, corresponding to graphite carbons. TEM image demonstrated that nanoparticles were successfully introduced to the active blend. Sodium alginate, sodium citrate, and TiO_2_ nanopowder were sonicated for 30 min and added dropwise into the mixture of CaCl_2_·2H_2_O, water, and ethanol to make TiO_2_ macro beads. The mixture of oxide beads and the active blend was stirred for 1 h and dried in air overnight to make oxide beads functionalized with the active blend. Alginate-based TiO_2_ macro beads, a photocatalyst for degradation of cationic pollutants, functionalized with the active blend showed better photocatalytic effect and adsorptive ability in mild conditions than the oxide beads functionalized with each component of the active blend or carbon dots prepared through energy-consuming hydrothermal synthesis. Functionalized TiO_2_ macro beads showed methylene blue (MB) dye adsorption capability of 82% and completely degraded MB in 30 min.

Ouyang et al. fabricated a nitrogen-doped carbon quantum dots/TiO_2_ (N-CDs/TiO_2_) composite from urea and tetrabutyl titanate (TBT) through hydrothermal treatment (Ouyang et al., 2020). The mixture of citric acid, urea, tetrabutyl titanate, ethylene glycol, and acetic acid was held in a Teflon-lined autoclave for 24 h at 200°C, centrifuged, washed with distilled water and ethanol, dried for 12 h at 60°C, and calcined for 1 h at 450°C in air to make N-CDs/TiO_2_. According to XRD results, (110) and (101) crystal facets of rutile appeared as N-CDs content increased. The SEM images showed that, as N-CDs content increased, the morphology of the N-CDs/TiO_2_ composite changed from nano sphere agglomerations, honeycomb, to empty spheres ((A) 4). The XPS result revealed that the elemental composition of N-CDs/TiO_2_-6 (mass ratio of urea to TBT = 6) was 71.96%, 2.6%, 18%, and 7.44% for C, N, O, and Ti, respectively. The photoluminescence (PL) spectra demonstrated that emission intensity decreased as N-CDs content increased. The N-CDs/TiO_2_ composite was used as a photocatalyst for the degradation of rhodamine B (RhB) under visible light. N-CDs/TiO_2_-1 degraded RhB in 120 min, which was 11.42 times better than pristine TiO_2_.

Li et al. prepared nitrogen and sulfur-containing carbon quantum dots (NCQDs)/TiO_2_ nanocomposites in a simple and eco-friendly way by depositing NCQDs onto TiO_2_ nanosheets (Li et al., 2018). Citric acid and thiourea were transferred to a Teflon-lined vial, heated in an 800 W microwave oven for 7 min, and subjected to dialysis for 1 day to make NCQDs. The TiO_2_ and NCQDs solution was sonicated for 30 min, stirred for 12 h at 80°C, washed with deionized water and ethanol, and lyophilized overnight to make NCQDs/TiO_2_. XRD pattern of NCQDs/TiO_2_ nanocomposite showed (101) peak of anatase and (110) peak of rutile. H-TEM images revealed that NCQDs/TiO_2_ nanocomposite exhibited (101) crystal planes of anatase, (111) crystal plane of rutile, and (101) plane of carbon. XPS result demonstrated that NCQDs/TiO_2_ nanocomposite contained C, O, N, S, and Ti. According to photoluminescence (PL) spectra, emission intensity decreased as NCQDs content increased. The NCQDs/TiO_2_ nanocomposite was used as a photocatalyst for the photoreduction of CO_2_ under simulated sunlight. The maximum amounts of CH_4_ and CO produced by the CO_2_ photoreduction using the nanocomposite in 1 h were 0.769 and 1.153 μmol, respectively, which were 7.79 and 7.61 times higher than those using only TiO_2_. The nanocomposite showed improved photocatalytic activity because NCQDs sensitized TiO_2_ to have enhanced light absorption.

Guo et al. prepared N and P co-doped carbon quantum dots (NP-CQDs) by simple heat treatment (Guo et al., 2018). Citric acid and phosphorylethanolamine were held in an autoclave with Teflon lining for 12 h at 200°C, centrifuged for 15 min at 10,000 rpm, and subjected to dialysis in a cellulose ester dialysis membrane for 1 day to make NP-CQDs. According to the TEM image, NP-CQDs had an average size of 3.03 ± 1.01 nm. The XPS result demonstrated that NP-CQDs contained C, O, N, and P. According to the photoluminescence (PL) spectroscopy, when 365 nm light is illuminated, the NP-CQDs solution emitted intense blue-green light with maxima at 475 nm. The NP-CQDs displayed high stability in saline conditions, quantum yield of 8.45%, and brilliant photostability. The NP-CQDs were used as sensitive and selective fluorescent probes for Fe^3+^. The NP-CQDs exhibited detection range from 0.05 to 200 μM. NP-CQDs and titanium butoxide were held in a Teflon-lined autoclave for 1 day at 160°C, centrifuged, washed with ethanol and water, and dried for 1 day at 60°C to make NP-CQDs/TiO_2_ nanocomposite. The NP-CQDs/TiO_2_ nanocomposite was used as a photocatalyst for the degradation of methylene blue (MB) under simulated solar light. The NP-CQDs/TiO_2_ nanocomposite had a degradation time of 15 min, which was shorter than pristine TiO_2_.

Midya et al. constructed titania nanoparticles (TiO_2_ NPs) and carbon dots (CDs) deposited polyvinyl imidazole cross-linked chitosan (cl-Ch-p (VI)/TiO_2_NPs-CDs) (Midya et al., 2020). The mixture of chitosan, acetic acid, potassium persulphate, 1-vinylimidazole, and diurethane dimethacrylate was held in a 600 W microwave oven for 2 min at 75°C to make cl-Ch-p (VI). The cl-Ch-p (VI) and titanium isopropoxide solution was held in the 750 W microwave oven for 2 min at 75 °C to make cl-Ch-p (VI)/TiO_2_NPs. The reaction mixture and sugar cane juice solution were treated with microwave for 4 min, precipitated using acetone, filtered using vacuum filtration, washed with acetone, and dried under vacuum at 50°C to make cl-Ch-p (VI)/TiO_2_NPs-CDs. XRD pattern of cl-Ch-p (VI)/TiO_2_NPs-CDs showed a peak at 24.1°, corresponding to (002) plane of carbon and (101) plane of anatase TiO_2_, and peaks at 37.5°, 47.7°, 53.9°, 62.6°, and 74.5°, corresponding to (004), (200), (105), (204), and (215) planes of crystalline TiO_2_ NPs, respectively. TEM image revealed that CDs and TiO_2_ NPs were introduced to the polymer matrix. XPS result demonstrated that cl-Ch-p (VI)/TiO_2_NPs-CDs contained C, N, O, and Ti. The cl-Ch-p (VI)/TiO_2_NPs-CDs nanocomposite was used as a photocatalyst for the degradation of organic materials under solar light. The cl-Ch-p (VI)/TiO_2_NPs-CDs nanocomposite showed degradation efficiency of ∼98.6%, ∼95.8%, and ∼98.2% for 2,4-dichlorophenol, reactive blue 4, and reactive red 15, respectively, which were better than those of cl-Ch-p (VI)/TiO_2_NPs and cl-Ch-p (VI)/CDs composites. The improved photocatalytic activity was due to the synergistic effects of CDs and TiO_2_ NPs and narrowed band gap.

Zhu et al. prepared a multiplecore@shells clustered carbon dots (CDs)/TiO_2_(P25)/reduced graphene oxide (rGO) nanocomposite through a simple hydrothermal process (Zhu W. et al., 2021). Citric acid and aphen were held in a Teflon-lined autoclave for 7 h at 200°C, centrifuged, dialyzed for 3 days, and subjected to freeze-drying under vacuum to make CDs. The mixture of P25, GO, and CDs solution was stirred for 12 h, held in the autoclave for 3 h at 120°C, washed with deionized water and absolute ethanol, and dried at 60°C overnight to make CDs/P25/rGO. XRD pattern of CDs/P25/rGO showed peaks at 25.3° and 27.5°, corresponding to (101) plane of anatase P25 and (110) plane of rutile P25 and a broad peak at 24.5°, indicating an intense interface interaction between components of CDs/P25/rGO. HTEM image of CDs/P25/rGO exhibited the (101) plane of P25. According to photoluminescence (PL) spectra, CDs/P25/rGO displayed a strong excitation-independent emission at 430 nm and a weak emission at 630 nm when the excitation wavelength was below 400 nm. EDS result revealed that the elemental composition of CDs/P25/rGO was 5.17%, 0.73%, 62.86%, and 31.24% for C, N, O, and Ti, respectively. Raman spectroscopy demonstrated D- and G-band at ∼1,361 and ∼1,601 cm^−1^, corresponding to disordered/defective and graphitic carbons, respectively. The CDs/P25/rGO nanocomposite was used as a high stability photocatalyst for the degradation of rhodamine-B (RhB), methylene blue (MB), and methyl orange (MO), and antibacterial performance under visible light. The 2% CDs/0.5% rGO/P25 composite showed the best photocatalytic activity. The rate constant of 2% CDs/0.5% rGO/P25 for Rh-B degradation was 29, 2.8, and 1.3 times higher than that of P25, rGO/P25, and CDs/P25, respectively. The CDs/P25/rGO nanocomposite displayed enhanced photocatalytic activity because CDs improved visible light-harvesting ability and promoted photogenerated charge separation.

Ratnayake et al. synthesized carbon-quantum-dot-decorated ZrO_2_ nanoparticles (CQDZ) by a simple method (Ratnayake et al., 2019). The colloids formed in the mixture of ZrOCl_2_·8H_2_O and ammonia were calcined for 4 h at 800°C to make ZrO_2_ nanoparticles. Ammonium citrate was held in a petri dish for 3 h at 180°C to make CQDs. Suspension of CQDs in absolute ethanol and 40 nm sized ZrO_2_ nanoparticles were mixed under sonication for 5 min and dried in air overnight to synthesize CQDZ. The TEM results showed that ZrO_2_ nanoparticles were mostly spherical with some rod-like particles. They also demonstrated that spherical CQDs were successfully adsorbed to ZrO_2_ nanoparticles. According to the XRD pattern, CQDZ exhibited peaks at 24.32°, 28.30°, and 34.52°, corresponding to (110), (020), and (120) planes of ZrO_2_ nanoparticles. The Raman spectrum of CQDZ confirmed the presence of G- and D-bands at 1,550 and 1,356 cm^−1^, corresponding to graphitic carbon and disordered carbon, respectively. CQDZ was used as a photocatalyst for the elimination of a dye, methylene blue (MB), in water under UV light. Using CQDZ, 95% of MB was eliminated after 1 h. Using pristine ZrO_2_ nanoparticles, however, only 34% of MB was eliminated after 1 h. The enhanced photocatalytic activity of CQDZ might be due to the CQDs effect of slowing down the electron-hole recombination.

**FIGURE 5:**
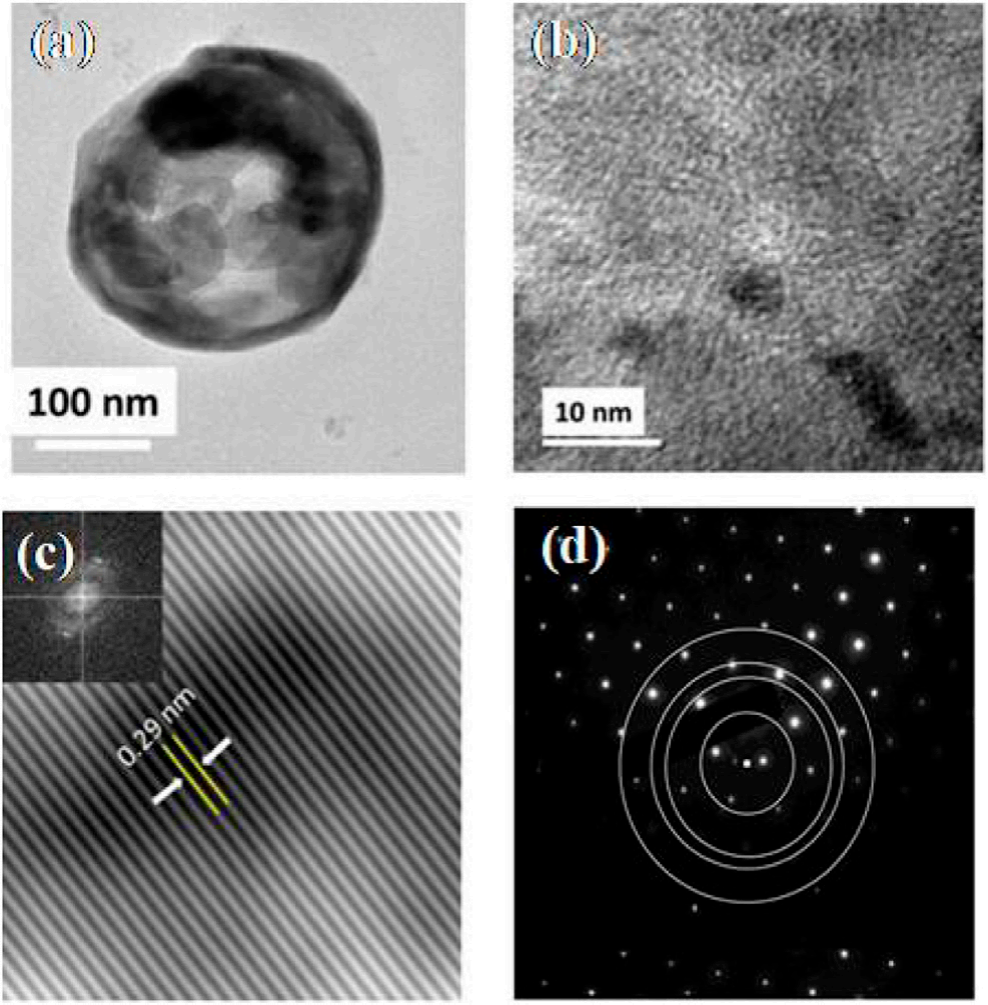
Field-emission transmission electron microscopic image of **(A)** BZO hollow nanospheres and **(B)** high-resolution transmission electron microscopic image of BZO hollow spheres. The inset to **(C)** is the fast Fourier transformed image of the highlighted portion in image **(B)**, **(C)** shows the inverse fast Fourier transformed image of the masked fast Fourier transformed image shown in the inset to **(C)**, **(D)** shows the selected area electron dispersion patterns of BZO. (Reproduced from Patra et al., 2018, American Chemical Society).

Patra et al. prepared carbon dots_BaZrO_3−δ_ (CDs_BZO) hybrid nanomaterials by loading CDs on BZO hollow nanospheres, which were both hydrothermally synthesized (Patra et al., 2018). Citric acid and ethylene diamine were held in a jacket for 5 h at 200°C, filtered with a 0.4 μm syringe filter, dialyzed with a dialysis bag against Milli-Q water for 1 day, and dried overnight at 80°C to make CDs. KOH, BaCl_2_·2H_2_O, and ZrOCl_2_·8H_2_O were held in an autoclave for 24 h at 200°C; centrifuged; washed with water, dilute acetic acid, and ethanol; and dried overnight in an electric oven at 100°C to make BZO. CDs and BZO were dispersed in ethanol for 2 h at 45°C by sonication to make CDs-BZO. Raman spectroscopy results exhibited D-band and G-band at 1,325 and 1,548 cm^−1^, corresponding to disordered carbon and graphitic carbon, respectively. According to the photoluminescence (PL) spectrum, CDs_BZO displayed a strong emission at 460 nm when the excitation wavelength was 340 nm. The FETEM images revealed that BZO were hollow spheres, and CDs were spheres with diameters of ∼2–7 nm shown in Figure 5. XPS result demonstrated that CDs_BZO contained C, N, O, Ba, and Zr. The CDs_BZO hybrid nanomaterial was used as a photocatalyst for H_2_ generation and methylene blue (MB) dye degradation under UV-visible light. The CDs_BZO with 3 wt% CDs had the highest hydrogen generation efficiency of 670 μmol/h/g with apparent quantum yield (AQY) of ∼4%, which was higher than AQY of BZO (∼2%). It also showed the highest MB degradation efficiency of ∼90%. Here, the defect states and oxygen vacancies generated by hydrothermal synthesis enable visible light absorption of the photocatalyst, and CDs improve the light absorption of the photocatalyst.

**SCHEME 2:**
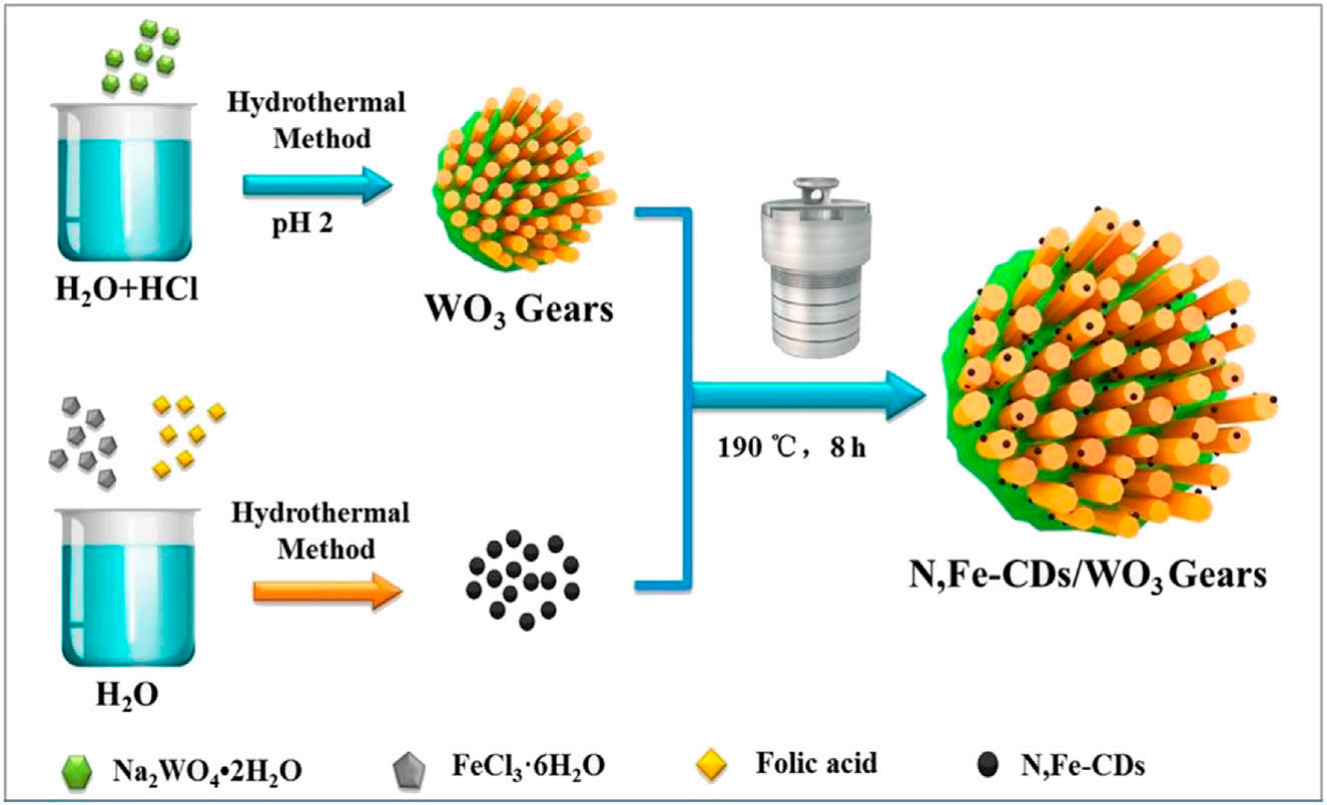
A schematic illustration of the fabrication of the gear-shaped WO3 (G-WO3) and N, Fe-CDs/G-WO3. (Reproduced from Ni et al., 2020, MDPI).

**FIGURE 6:**
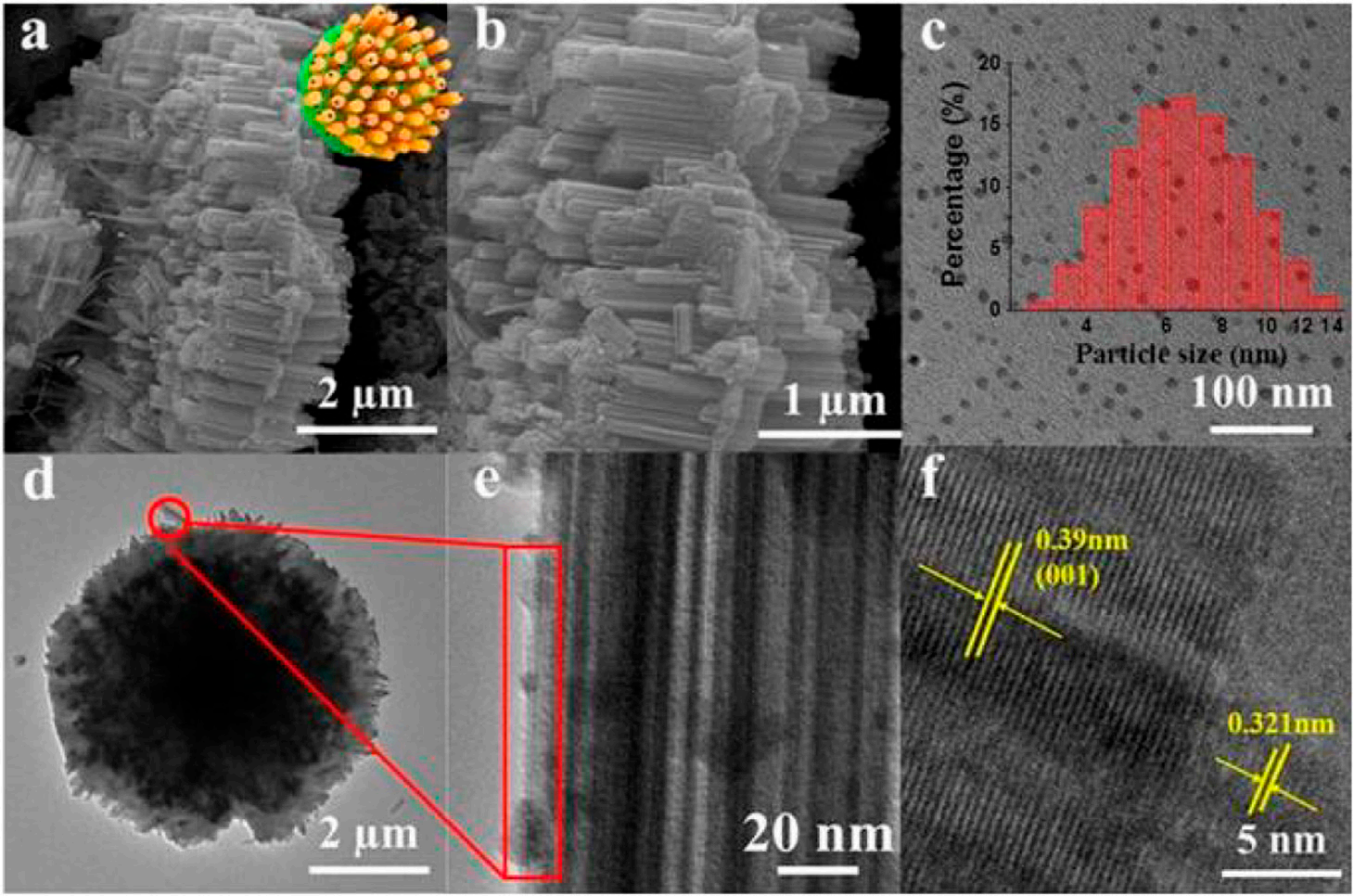
SEM and TEM of N,Fe-CDs; N,Fe-CDs/G-WO3-0.6 composite. **(A,B)** SEM of N,Fe-CDs/G-WO3-0.6; **(C)** TEM of N,Fe-CDs with the inset for the size distribution; **(D)** TEM of N,Fe-CDs/G-WO3-0.6; **(E,F)** HRTEM of N,Fe-CDs/G-WO3-0.6. (Reproduced from Ni et al., 2020, MDPI).

Ni et al. synthesized nitrogen and iron co-doped carbon dots/gear-shaped WO_3_ (N,Fe-CDs/G-WO_3_) composite by simple hydrothermal synthesis (Ni et al., 2020). Na_2_WO_4_·2H_2_O solution was held in a Teflon-lined autoclave for 12 h at 180°C, washed with deionized water and ethanol, and dried for 6 h in an oven at 80°C to make G-WO_3_. Folic acid and FeCl_3_·6H_2_O were held in the autoclave for 4 h at 200°C, filtered with 0.22 μm aqueous microporous membranes, dialyzed for 6 h, and subjected to freeze-drying to make N,Fe-CDs. N,Fe-CDs and G-WO_3_ were held in the autoclave for 8 h at 190°C, centrifuged, and dried at 80°C to make the N,Fe-CDs/G-WO_3_ composite in Scheme 2. SEM and TEM images of G-WO_3_ showed in Figures 6A–F the 3-D gear-like structure consisting of a plate with many protruding nanorods on its surface. The particle diameters was N-Fe-CDs ranged from 3.5 to 13.5 nm, with an average diameter of 6.5 nm. HTEM images showed that G-WO_3_ exhibited lattice fringe with a lattice spacing of 0.39 nm and N,Fe-CDs exhibited lattice fringe with a lattice spacing of 0.32 nm. The XRD pattern of N,Fe-CDs/G-WO_3_ revealed peaks at 13.8°, 22.6°, 28.0°, 36.3°, and 49.7°, corresponding to the hexagonal crystalline phase of G-WO_3_. XPS result demonstrated that N,Fe-CDs/G-WO_3_ contained C, N, O, W, and Fe. According to the photoluminescence (PL) spectrum, PL intensity of N,Fe-CDs/G-WO_3_ was reduced after loading of N,Fe-CDs. The N,Fe-CDs/G-WO_3_ composite was used as a photocatalyst for degradation of rhodamine B (RhB) under UV-vis-NIR light. 81.4%, 97.1%, and 75% of RhB were degraded in 2 h under UV, visible, and NIR light, respectively, which were better than those of commercial WO_3_, G-WO_3_, and N-CDs/G-WO_3_ composites. The composite was also used for the degradation of methylene blue (MB), ciprofloxacin (CIP), tetracycline hydrochloride (TCH), and oxytetracycline (OTC) under visible light. 91.1%, 70.5%, 54.5%, and 47.8% of respective materials were degraded in 3 h. According to the radical trapping experiments, the conversion between Fe (III) and Fe (II) is the most important factor in photocatalysis. The photocatalytic activity was improved because N,Fe-CDs enhanced light absorption ability, and the gear shape of the WO_3_ increased light reflections.

Jamila et al. constructed a WO_3_/graphene oxide/N-doped carbon quantum dots (WO_3_/GO/NCQDs) composite by introducing NCQDs to GO modified WO_3_ nanosheets (Jamila et al., 2020a). Graphite powder, sodium nitrate, concentrated sulfuric acid, potassium permanganate, and H_2_O_2_ were stirred with a magnetic stirrer, filtered, washed with HCl, centrifuged, and dried at 60°C in an oven to make GO. Sodium tungstate and nitric acid were mixed under magnetic stirring for 10 min, kept for 6 h at 40°C, washed using distilled water and ethanol, dried at 60°C, and calcined for 4 h at 600°C to make WO_3_. Citric acid and urea were held in a Teflon-lined autoclave for 4 h at 160°C and NCQDs. TEM image revealed that GO and NCQDs were fixed on WO_3_ nanosheets. EDAX result demonstrated that the elemental composition of WO_3_/GO/NCQDs was 9.50, 43.03, 30.04, and 17.43% for W, O, C, and N, respectively. Photoluminescence (PL) intensity of WO_3_/GO/NCQDs decreased when NCQDs content increased. According to Raman spectroscopy, WO_3_/GO/NCQDs displayed D- and G-band at 1,435 and 1,575 cm^−1^, corresponding to disordered carbon and graphitic carbon, respectively. The WO_3_/GO/NCQDs composite was used as a photocatalyst for the degradation of methyl orange (MO) under visible light irradiation. The WO_3_/GO/NCQDs composite with 1.5 ml NCQDs showed the best degradation efficiency of 86%. The WO_3_/GO/NCQDs composite exhibited better photocatalytic activity than WO_3_/GO composite and WO_3_. The photocatalytic effect was improved because NCQDs extended the absorption region to the visible light region, increased active sites, and promoted charge separation.

Velumani et al. constructed carbon quantum dots supported ZnO hollow spheres (ZnO/C-dots) by a solvothermal process (Velumani et al., 2020). Glucose and urea were added to 250°C copra oil, and the mixture was stirred for 3 min. Chloroform was added to the solution, and the upper oil layer was removed by a separation funnel to make C-dots. Zinc acetate dihydrate, urea, polyethylene glycol-400, and C-dots solution were magnetically stirred at room temperature for 90 min, held in a Teflon-lined autoclave for 15 h at 180°C, centrifuged, washed with ethanol and deionized water, and dried at 80°C in an oven for 12 h to make ZnO/C-dots. The photoluminescence (PL) spectra showed that ZnO/C-dots exhibited the strongest emission when the excitation wavelength was 345 nm. PXRD pattern of ZnO/C-dots revealed peaks at 31.8°, 34.4°, 36.3°, 47.5°, 56.6°, 62.9°, and 68.0° corresponding to (100), (002), (101), (102), (110), (103), and (112) planes of ZnO, respectively. According to the TEM result, ZnO/C-dots were spheres with the size of 500 nm. The ZnO/C-dots nanocomposite was used as a photocatalyst for the degradation of methylene blue (MB) under UV-vis light. Using the ZnO/C-dots nanocomposite, 96% of MB was degraded in 30 min. However, using pristine ZnO, only 63% of MB was degraded in 30 min. The photocatalytic effect was improved because C-dots slowed down the photogenerated electron-hole pairs’ recombination rate and enhanced visible light absorption. The nanocomposite could be used for five cycles, showing high stability.

Omer et al. prepared a ZnO-phosphorous and nitrogen co-doped carbon quantum dots (CDs) nanocomposite in an eco-friendly way (Omer et al., 2018). Zn(CH_3_COO)_2_·2H_2_O in methanol was adjusted to pH 8 using NaOH, held in a Teflon-lined autoclave for 6 h at 200°C, washed with methanol, filtered, and dried at 60°C in an oven to make the ZnO nanoparticles. Grounded coffee and concentrated phosphoric acid were kept in a water bath for 30 min at 90–95°C, filtered, centrifuged for 30 min at 14,000 rpm, adjusted to pH 1–3, and dialyzed with dialysis membrane for 5 h to make CDs. ZnO and CDs were stirred for 2 h to make the ZnO-CDs nanocomposite. The TEM result showed that CDs and ZnO had diameters of 3–4 nm and 15–20 nm. The XRD pattern of ZnO-CDs nanocomposite displayed characteristic peaks of both ZnO and CDs. According to the XPS result, CDs contained C, O, N, and P.

The excitation-independent fluorescence of CDs centered around 480 nm was confirmed by fluorescence spectrum. The ZnO-CDs nanocomposite was used as a photocatalyst for the degradation of organic materials under energy-efficient weak LED light (visible-NIR light) irradiation. The ZnO-CDs nanocomposite exhibited methylene blue (MB) degradation efficiency of 80% in 10 h, which was much higher than that of pristine ZnO (10%). The photocatalytic activity was enhanced because CDs promoted photogenerated charge separation and transfer, and CDs’ up-conversion properties improved light absorption.

El-Shamy et al. developed a carbon quantum dots/zinc peroxide (C_Dots_/ZnO_2_) composite by introducing C_Dots_ to ZnO_2_ nanoparticles (El-Shamy A.g., 2020). d-Glucose solution was heated in a 500 W microwave oven for 7 min and centrifuged to make C_Dots_. Zinc acetate solution, 30% H_2_O_2_, NaOH, and ethanol were heated in a 400 W microwave oven for 3h, filtered, washed using DI water, and dried at 60°C in an oven overnight to make ZnO_2_. The mixture of C_Dots_ solution and ZnO_2_ solution was stirred for 30 min at room temperature, centrifuged, washed using ethanol, and air-dried to make C_Dots_/ZnO_2_ composite. The XRD result of C_Dots_/ZnO_2_ composite showed a hump at 23°, corresponding to the (002) plane of graphitic carbon, and peaks corresponding to (111), (200), (220), and (311) planes of crystalline ZnO_2_. The TEM image of C_Dots_/ZnO_2_ composite revealed the (110) plane of graphitic C_Dots_ and (111) plane of ZnO_2_. The energy dispersive spectroscopy (EDS) result demonstrated that C_Dots_/ZnO_2_ composite contained C, O, and Zn. According to the up-conversion photoluminescence (PL) spectra, C_Dots_ absorb NIR light and emit light with a shorter wavelength. The C_Dots_/ZnO_2_ composite was used as a photocatalyst for the degradation of methyl orange (MO), methylene blue (MB), and rhodamine B (RhB) under UV-A light irradiation. The C_Dots_/ZnO_2_ composite exhibited better degradation efficiencies for MO (91 ± 2% in 60 min), MB (99 ± 1% in 50 min), and RhB (99 ± 1% in 80 min) than ZnO_2_, TiO_2_, and C_Dots_/TiO_2_ due to promoted charge separation by C_Dots_ and ZnO_2_, electron capturing and up-conversion photoluminescence by C_Dots_, and the C_Dots_/ZnO_2_ structure advantageous to charge transfer and separation. The C_Dots_/ZnO_2_ composite could be used five times without any decrease in photocatalytic performance.

El-Shamy et al. developed polyvinyl alcohol/carbon dot-decorated zinc peroxide (PVA/CZnO_2_) films by introducing CZnO_2_ to PVA (El-Shamy A. G., 2020). The microwave irradiation method was used to make carbon dots. The mixture of Zn(CH_3_COO)_2_∙2H_2_O, triple-distilled water, H_2_O_2_, NaOH, and ethanol was held in a 400 W microwave oven for 3 h at 180°C, filtered, washed with water, and dried for 1 day at 60°C in an oven to make ZnO_2_. ZnO_2_ solution and carbon dot solution were mechanically stirred for 1 h, centrifuged, filtered, washed with ethanol, and dried in air to make CZnO_2_. PVA and triple-distilled water were stirred at 67 ± 3°C for 2 h, transferred to glass dishes, and air-dried for 3 days at ambient temperature to make PVA. CZnO_2_ was dispersed in PVA solution, transferred to glass dishes, and air-dried for 3 days to make PVA/CZnO_2_. XRD pattern of PVA/CZnO_2_ exhibited a peak at 19.5° for (101) plane of PVA and peaks of ZnO_2_. FE-SEM images showed that CZnO_2_ particles were uniformly dispersed on PVA matrix.

According to Brunauer–Emmett–Teller (BET) analysis, the PVA/CZnO_2_ nanocomposite had a large surface area of 241.04 m^2^ g^−1^, larger than the PVA/ZnO_2_ nanocomposite (167.17 m^2^ g^−1^). The PVA/CZnO_2_ nanocomposite with 4 wt% CZnO_2_ exhibited an adsorption capacity of 1,972 ± 40 mg/g against methylene blue (MB), which was higher than that of the PVA/ZnO_2_ nanocomposite with 4 wt% ZnO_2_ (1,831 ± 20 mg/g). At pH = 6, room temperature, with low ionic strength, 40 mg of PVA/CZnO_2_ nanocomposite eliminated 98% of 2,000 mg/L MB in 60 min. The enhanced adsorption ability came from improved electrical conductivity, crystallinity, pore size, and mechanical properties. A solution with pH = 1 removed 75% of MB from the nanocomposite, and the nanocomposite could be used five times with only a slight decrease in adsorption efficiency (∼2%).

El-Shamy et al. constructed polyvinyl alcohol/carbon quantum dots (PVA/CQDs) nanocomposite films using the solution casting method (El-Shamy and Zayied, 2020). PVA and bi-distilled water were mechanically stirred at 65 ± 3°C for 2 h, transferred to a petri-dish, and air-dried for 3 days to make PVA films. The mixture of d-glucose, acetone, and bi-distilled water was heated in a 700 W microwave oven for 13 min and centrifuged to make CQDs. The mixture of PVA solution and CQDs solution was mechanically stirred and dried for 3 days at ambient temperature to make PVA/CQDs. XRD pattern of PVA/CQDs showed a broad peak at 19.5°, corresponding to the crystalline part of semi-crystalline PVA. According to photoluminescence (PL) spectra, as CQDs content increased, PL intensity increased and PL peak exhibited red shift behavior. FE-SEM images revealed that, as CQDs content increased, the surface area of PVA/CQDs increased. The PVA/CQDs nanocomposite films were used for the elimination of methylene blue (MB) from waste water by adsorption at room temperature. The 80 mg of (PVA/CQDs 2 wt%) film eliminated 97 ± 1% of 30 mg/L MB in 40 min at pH = 12. In the basic environment, -OH groups in PVA and -OH groups or -COOH groups in CQDs were ionized, making the composite surface more negatively charged. This made the composite more easily adsorb cationic MB. The PVA/CQDs nanocomposite films could be used five times after ethanol treatment without any photocatalytic activity decrease.

Nayak et al. fabricated carbon quantum dots cross-linked polyvinyl pyrrolidone (PVP-CD) hydrogels (Nayak et al., 2020). Lemon juice and cysteamine were evaporated in a beaker at 90°C, held in a Teflon-lined autoclave for 6 h at 150°C, diluted with water, centrifuged at 10,000 rpm, and filtered by a 0.2 μm syringe filter to make CD. PVP was treated with NaOH under reflux condition at 140°C for 2 days, further treated with formaldehyde for 1 h at 0°C and pH 9, finally treated with NaBH_4_ under stirring at ambient temperature for 1.5 h, and dialyzed for 6 h to make carboxylate-PVP. The mixture of carboxylate-PVP, EDC, MES buffer, and NHS was dialyzed with water for 18 h, concentrated at 60°C in a vacuum, treated with water, and held in an air oven at 75°C to make PVP-CD. For PVP-CD, storage modulus was higher than loss modulus, indicating the elasticity of PVP-CD. PVP-CD emitted blue fluorescence when UV light was illuminated. The SEM image showed that cross-linking with CD has increased the surface area of PVP-CD. 50 mg of PVP-CD hydrogel adsorbed ∼2 mg of malachite green (MG), crystal violet (CV), and eosin Y in 12 h, which followed pseudo-second-order kinetics. The good adsorptive ability came from hydrogen bonding, inductive effect, and π-π interaction of both CD and the PVP backbone. The PVP-CD hydrogel also showed excellent photodegradative capability of dyes under solar light by degrading them in 30 min, which followed pseudo-first-order kinetics. The PVP-CD hydrogel killed gram-positive and gram-negative bacteria in 10 min under solar light. The degradation of dyes and bacterial elimination were all carried out by reactive oxygen species generated from CD. The PVP-CD hydrogel could be used four times when washed with dilute acid.

Gong et al. fabricated carbon quantum dots (CDs) from wood powder *via* hydrothermal process (Gong et al., 2019). The mixture of wood powder, citric acid, ethanediamine, and deionized water was sonicated for 20 min, magnetically stirred for 30 min, held in a Teflon-lined autoclave for 6 h at 200°C, vacuum-filtrated, heated for 12 h at 30°C, washed using anhydrous ethanol, dried for 12 h at 30°C, dissolved in deionized water, and filtrated with membrane to make CDs. The XPS result showed that CDs contained C, O, and N. The CDs were environment-friendly, nontoxic, and water-soluble. According to photoluminescence (PL) spectra, the CDs solution emitted blue light under UV light irradiation with a quantum yield of 47.4%. Furthermore, the CDs solution was used as a fluorescent stamp-pad and pen ink, showing its possibility in anti-counterfeit applications. The mixture of cerium nitrate, deionized water, and dilute ammonia was held in an autoclave for 4 h at 150°C, centrifuged for 5 min at 8,000 rpm, washed by ethanol and deionized water, dried for 1 day at 80°C, and calcined for 4 h at 450°C to make CeO_2_. CDs solution, CeO_2_ powder, and deionized were magnetically stirred for 10 h, centrifuged, washed by ethanol and water, and dried for 12 h at 90°C to make CDs/CeO_2_ composite. The HTEM image revealed that CDs were successfully incorporated into CDs/CeO_2_ composite. The XRD patterns demonstrated peaks at 28.5, 33.2, 47.5, 56.3, and 59.1° corresponding to <111>, <200>, <220>, <311>, and <400> planes of cubic fluorite CeO_2_. Raman spectroscopy showed an F2g mode of fluorite phase at 430 cm^−1^, a D-band at 1,410 cm^−1^, corresponding to lattice defect of carbon, and a G-band at 1,580 cm^−1^, corresponding to graphitic carbon. CDs/CeO_2_ composite was used as a photocatalyst for the degradation of methylene blue (MB) under visible light. The reaction constant of CDs/CeO_2_ composite was much higher than that of CeO_2_.

Jamila et al. prepared copper oxide/N-doped carbon quantum dots (CuO/NCQDs) by introducing NCQDs into CuO nanoleaves (Jamila et al., 2020b). Copper nitrate, distilled water, and NaOH were stirred, washed with distilled water, centrifuged, dried at 60°C, and calcined for 2 h at 500°C to make CuO. Citric acid, deionized water, and urea were held in an autoclave for 4 h at 180°C and centrifuged for 10 min at 4,000 rpm to make NCQDs. CuO, distilled water, and NCQDs were magnetically stirred and dried at 60°C in an oven to make CuO/NCQDs. XRD pattern of CuO/NCQDs exhibited peaks at 32.3°, 35.2°, 38.3°, 48.8°, 53.2°, 57.9°, 61.3°, 66.0°, 67.9°, and 75.2° corresponding to (110), ( $\bar{1}$ 11), (111), ( $\bar{2}$ 02), (020), (202), ( $\bar{1}$ 13), (022), (220), and (203) planes of CuO and a broad carbon peak around 30°. According to the TEM image of CuO/NCQDs, NCQDs were dispersed on slightly distorted CuO nanoleaves. The EDAX result demonstrated that the elemental composition of CuO/NCQDs was 34.31%, 50.45%, 6.22%, and 9.02% for Cu, O, C, and N, respectively. According to PL results, the CuO/NCQDs had lower photoluminescence intensity than CuO, showing decreased electron-hole recombination. The CuO/NCQDs were used as a photocatalyst for the degradation of methyl orange (MO) under sunlight. The CuO/NCQDs with 1.5°ml of NCQDs displayed the highest degradation efficiency (92.0%). The CuO/NCQDs exhibited better degradation efficiency than pristine CuO because NCQDs increased charge separation efficiency and visible light-harvesting capacity.

Qu et al. fabricated a carbon quantum dots (CQDs)/KNbO_3_ composite in mixed-calcination and hydrothermal routes by anchoring CQDs on KNbO_3_ (Qu et al., 2018). The mixture of distilled water, Nb_2_O_5_, and KOH was held in a Teflon-lined autoclave for 12 h at 160°C, washed by ethanol and distilled water, dried overnight at 80°C, and calcined for 1 h at 400°C to make KNbO_3_ particles. The mixture of triple-distilled water, l-ascorbic acid, and glycol was held in the autoclave for 70 min at 160°C to make CQDs. KNbO_3_ particles were dispersed in CQDs solution, stirred for 30 min at ambient temperature, and dried for 8 h at 80°C to make CQDs/KNbO_3_ composite. According to photoluminescence (PL) spectra, when excitation wavelength was 400–500 nm, CQDs exhibited down-conversion PL. When excitation wavelength was 500–600 nm, CQDs exhibited up-conversion PL. The TEM result showed that CQDs were well dispersed on the surface of KNbO_3_ particles. EDX result revealed that the elemental composition of CQDs/KNbO_3_ composite was 19.40%, 23.26%, 21.89%, and 35.45% for C, Nb, K, and O, respectively. The CQDs/KNbO_3_ composite was used as a photocatalyst for the degradation of crystal violet dye and hydrogen evolution under visible light. When the mass ratio was CQDs: KNbO_3_ = 1.5:0.5, the dye degradation efficiency and hydrogen evolution rate were 70.00% and 468.72 μmol/h/g, which were higher than those of pristine KNbO_3_ (41.50% and 245.52 μmol/h/g). The photocatalytic performance was enhanced because CQDs converted visible light to UV light, which could activate KNbO_3_, and captured electrons to help the separation of electron-hole pairs. The CQDs/KNbO_3_ composite could be used four times without a significant decrease in performance.

**FIGURE 7:**
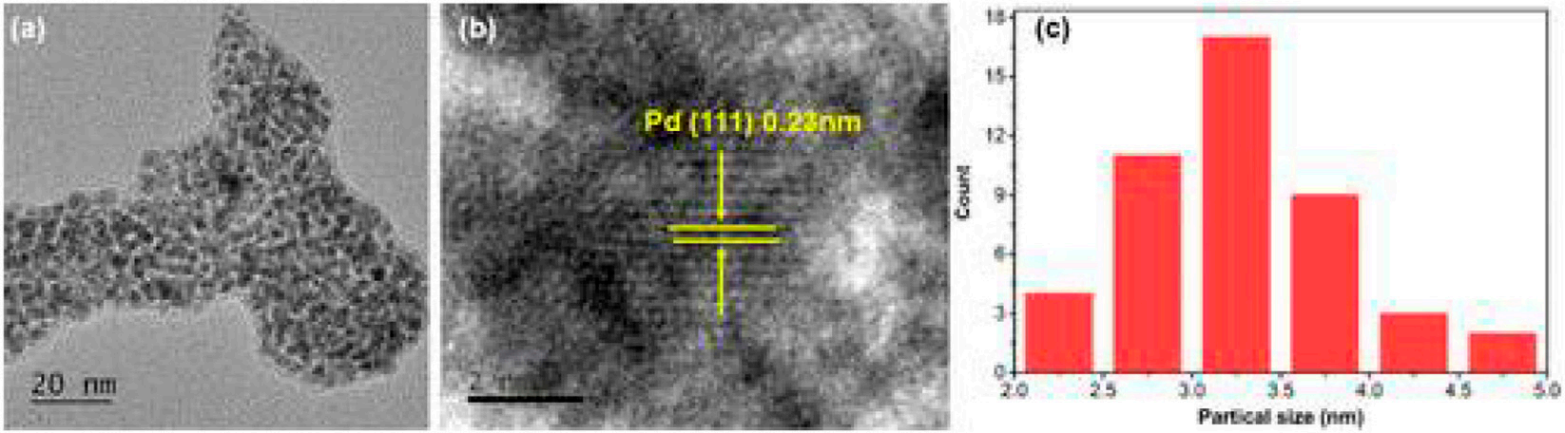
**(A)** TEM and **(B)** HR-TEM images of Pd@CD-CONH and **(C)** histogram. (Reproduced from Selim et al., 2020, American Chemical Society).

**FIGURE 8:**
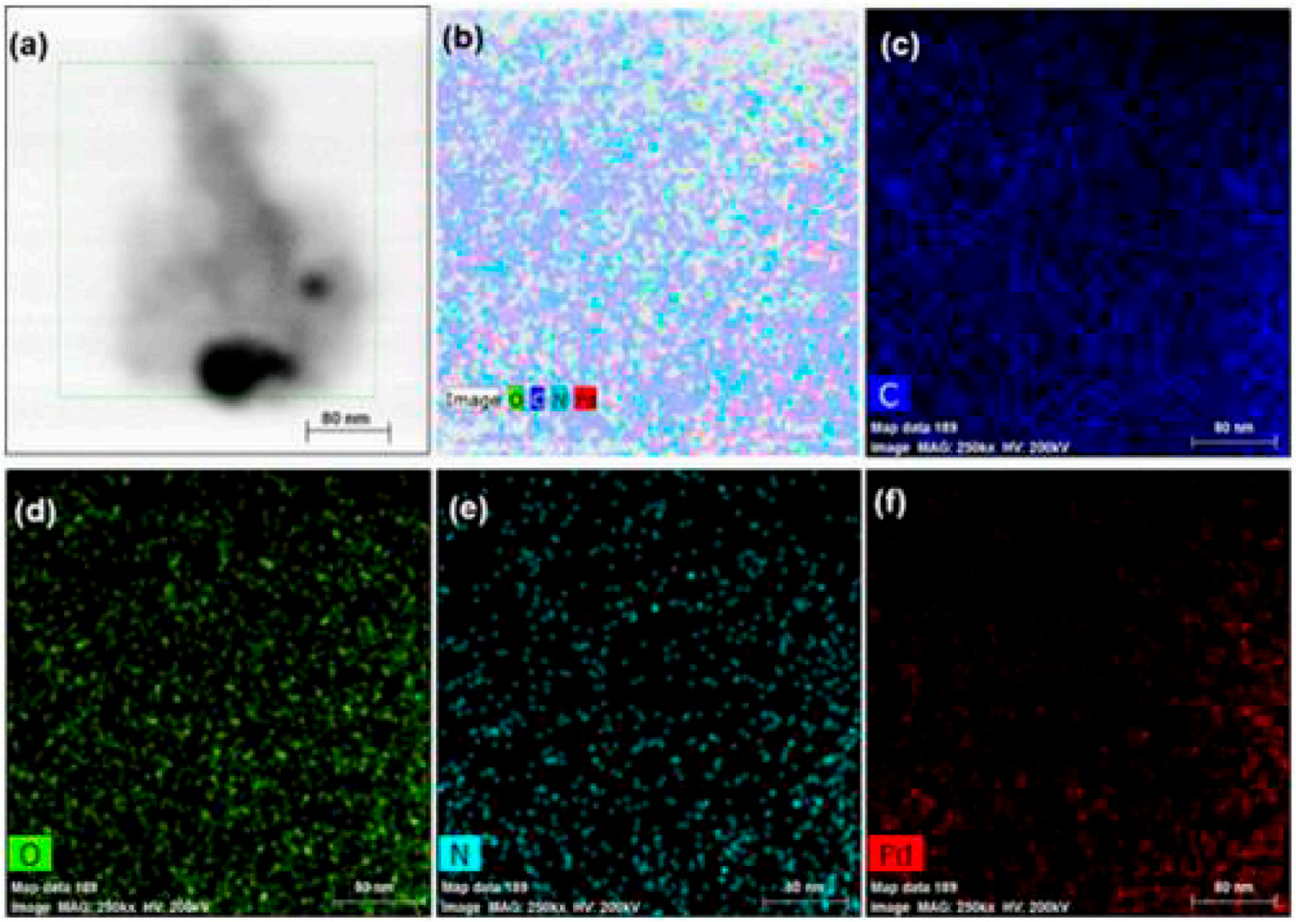
**(A)** TEM images of Pd@CD-CONH, **(B)** EDAX mapping of some areas of the catalyst, **(C)** C, **(D)** O, **(E)** N, and **(F)** Pd. (Reproduced from Selim et al., 2020, American Chemical Society).

Selim et al. synthesized a Pd-doped hybrid nanocatalyst (Pd@CD-CONH) by cross-linking carbon dots (CDs) with benzene-1,4-diamine (BDA) and doping the resulting polymer with palladium nanoparticles (Pd NPs) (Selim et al., 2020). The mixture of CD-COCl, dry THF, Et_3_N, and BDA was dried at 40°C under vacuum; transferred to a sintered funnel; washed with water, hexane, and diethyl ether; dissolved in methanol, and dried again at 40°C under vacuum to make CD-CONH. The mixture of CD-CONH, ethanol, and PdCl_2_ was dried at 40°C under vacuum, transferred to a sintered funnel, washed with water, and dried for 12 h at 60°C in a vacuum oven to make Pd@CD-CONH. The PXRD patterns showed peaks corresponding to (100), (111), (200), (220), (311), and (222) planes of Pd NPs. According to the HTEM result, Pd@CD-CONH exhibited lattice fringes of the (111) plane (Figure 7). The Pd peak is observed in the EDX mapping of Pd@CDCONH along with C, N, and O, indicated that the Pd NPs have been clearly homogeneously distributed throughout the entire CD polymer (Figure 8). The XPS result revealed that Pd@CD-CONH contained C, N, and Pd. The Pd@CD-CONH was used as a recyclable sonocatalyst for degrading rhodamine B dye in water without using any light. With Pd@CD-CONH, 99% of rhodamine B dye was degraded in 5 min in dark, which was faster than other catalysts. Mechanistic studies indicate that reactive oxygen species (ROS) produced with the help of CDs and Pd NPs enhance the sonocatalytic effect.

He et al. synthesized a MIL-53(Fe)/carbon quantum dots/noble metal (MIL-53(Fe)/CQDs/MNPs) photocatalyst in a simple way (He et al., 2021). The mixture of water, MIL-53(Fe), and CQDs was stirred for 10 min at 500 rpm, centrifuged, washed, and dried in a vacuum for 1 day at 60°C to make MIL-53(Fe)/CQDs. HAuCl_4_, AgNO_3_, or H_2_PdCl_4_ was added to the mixture of water, ethanol, and MIL-53(Fe)/CQDs, illuminated with 300 W xenon lamp for 60° min, treated with N_2_, washed with ethanol, and blow-dried by N_2_ to make MIL-53(Fe)/CQDs/MNPs. The zeta potential of CQDs (−14.8°mV) indicated a negative charge, and the zeta potential of MIL-53(Fe) (4.6 mV) indicated a positive charge. Therefore, CQDs and MIL-53(Fe) were combined readily by electrostatic attraction. XRD pattern of MIL-53(Fe)/CQDs/Au showed peaks corresponding to MIL-53(Fe), a weak peak at 26° corresponding to carbon, and a weak peak at 38.1° corresponding to (111) crystal planes of Au. TEM images revealed that noble metal particles were dispersed on MIL-53(Fe)/CQDs. XPS result demonstrated that MIL-53(Fe)/CQDs/MNPs contained C, O, Fe, and noble metal. With the MIL-53(Fe)/CQDs/2%Au (mass ratio of Au to MIL-53(Fe)/CQDs = 2%) photocatalyst, almost 100% of Cr (VI) was reduced in 20 min under visible light irradiation. The MIL-53(Fe)/CQDs/2%Au displayed a rate constant of 0.1820 min^−1^, which was about 7.4, 2.76, 1.77, and 1.47 times higher than that of MIL-53(Fe), MIL-53(Fe)/CQDs, MIL-53(Fe)/CQDs/2%Ag, and MIL-53(Fe)/CQDs/2%Pd. It was revealed that the MIL-53(Fe)/CQDs/2%Au could also eliminate Cr (VI) and dyes at the same time. The enhanced photocatalytic activity came from increased light absorption, the high electrical conductivity of CQDs and gold, and the surface plasmon resonance effect of gold.

Zhang et al. prepared carbon quantum dots (CQDs) modified graphitic carbon nitride (CCN) in a simple way (Zhang L. et al., 2020). Citric acid was heated for 20 min at 180°C, treated with NaOH, sonicated for 20 min, centrifuged for 10 min at 8,000 rpm, and dialyzed for 2°days using a dialysis bag to make CQDs. The mixture of urea and CQDs solution was held in an alumina crucible for 2 h at 550°C, stripped in HNO_3_ solution for 1 h at 80°C, diluted using deionized water, treated with NaOH, centrifuged for 5 min at 8,000 rpm, and washed with water to make CCN. The SEM images with element mapping showed that CQDs were uniformly distributed on graphitic carbon nitride. XRD pattern of CCN exhibited peaks at 13.1° and 27.5°, corresponding to <002> and <100> crystal planes of graphite. XPS result revealed that CCN contained C, N, and O. The CCN was used as a photocatalyst for the degradation of methylene blue (MB) and rhodamine B (RhB) and hydrogen evolution under visible light irradiation. The 4CCN (0.98 wt% CQDs) showed the shortest MB degradation time of 20 min and the highest hydrogen evolution rate of 1,291 μmol/h/g. The CCN had better performance than pristine graphitic carbon nitride because CQDs increased visible light absorption and promoted photogenerated charge separation and transfer.

Seng et al. developed a nitrogen-doped carbon quantum dots-decorated 2D graphitic carbon nitride (NCQD/g-C_3_N_4_) composite through a hydrothermal method (Seng et al., 2020). The mixture of distilled water, citric acid, and urea was held in a Teflon-lined autoclave for 4 h at 150°C, centrifuged for 20 min at 12,000 rpm, and dried at 90°C overnight in an oven to make bulk NCQDs. Urea was held in a covered porcelain crucible for 3 h at 550°C and ground into powder to make bulk g-C_3_N_4_. g-C_3_N_4_ exfoliated by sonication and NCQDs solution were vigorously stirred for 30 min at ambient temperature, held in the autoclave for 4 h at 120°C, filtered by vacuum filter, washed by distilled water, and dried at 70°C in an oven overnight to make NCQD/g-C_3_N_4_. According to the TEM result, the morphology of g-C_3_N_4_ did not change after the addition of NCQDs. EDX result showed that the elemental composition of NCQD/g-C_3_N_4_ was 31%, 18.85%, 0.58%, and 49.5% for C, N, O, and Si, respectively. The XRD pattern of NCQD/g-C_3_N_4_ exhibited peaks at 13.2° and 27.4°, corresponding to (100) and (002) planes of graphite. Photoluminescence (PL) spectrum revealed that PL intensity of NCQD/g-C_3_N_4_ decreased after NCQDs loading. The NCQD/g-C_3_N_4_ composite was used as a photocatalyst for the degradation of methylene blue (MB) under LED light. The 1°wt% NCQD/g-C_3_N_4_ (1°wt% NCQD) exhibited the highest MB degradation efficiency of 54.6%, which was 2.6 times higher than that of pristine g-C_3_N_4_. The 1°wt% NCQD/g-C_3_N_4_ displayed an MB degradation rate constant of 5.061 × 10^−3^ min^−1^, which was 3.85 times higher than that of pure g-C_3_N_4_. The improved photocatalytic activity was due to increased speed of charge transfer, suppressed electron-hole recombination, and optimized heterojunction interface between g-C_3_N_4_ and NCQD. When the 1wt% NCQD/g-C_3_N_4_ was used for thee times, the degradation efficiency was 91.2% of original efficiency, showing high stability of the composite.

He et al. constructed a 3D graphene oxide- (GO-) carbon quantum dots/g-C_3_N_4_ nanosheet (GA-CQDs/CNN) aerogel by a simple hydrothermal method (He H. et al., 2018). EDTA-2Na•2H_2_O was held in a quartz boat for 2 h at 350°C, dissolved in ethanol, centrifuged for 30 min at 12,000 rpm, and freeze-dried overnight to make CQDs. Dicyandiamide underwent thermal polycondensation to make bulk g-C_3_N_4_. The mixture of deionized water, bulk g-C_3_N_4_, and H_2_SO_4_ was sonicated for 8 h and washed by distilled water to make CNN. Graphite powder underwent modified Hummers’ method to make GO. The mixture of CQDs and CNN was held in an autoclave for 4 h at 180°C, mixed with ultrasonic GO, stirred for 1°h, heated again for 6 h at 180°C, washed by distilled water, and freeze-dried overnight to make GA-CQDs/CNN. The XRD patterns of GA-CQDs/CNN exhibited peaks at 27.85° and 13.29°, corresponding to (002) and (100) planes of pristine CNN. XPS result showed that GA-CQDs/CNN contained C, O, and N. HTEM image revealed a distinguished interface between reduced GO and CNN. According to photoluminescence (PL) spectra, GA-CQDs/CNN displayed the PL quenching phenomenon. The GA-CQDs/CNN was used as a photocatalyst for the degradation of methyl orange (MO) under visible light irradiation. The GA-CQDs/CNN-24% (mass ratio of GO to CNN = 24%) exhibited MO degradation efficiency of 91.1%, which was 7.6 times higher than that of bulk g-C_3_N_4_. The GA-CQDs/CNN also displayed high adsorption capacity. The photocatalytic and adsorptive performance was improved because the large specific surface area of the porous structure was advantageous for light absorption and material adsorption. Moreover, the large planar interface between CNN and GO promoted photoexcited charge transfer and separation, and CQDs enhanced light absorption and charge separation. The GA-CQDs/CNN could be used for four cycles without a significant decrease in performance.

Miao et al. synthesized an all-solid-state Z-scheme graphitic carbon nitride (g-C_3_N_4_)/Ag_3_PO_4_/nitrogen-doped carbon dots (NCDs) photocatalyst by a simple solution process by anchoring NCDs onto g-C_3_N_4_/Ag_3_PO_4_ photocatalyst (Miao et al., 2018a). The mixture of water, citric acid, and ethylene diamine was held in a Teflon-lined autoclave for 5 h at 200°C, dialyzed for 1°day, freeze-dried, and dispersed in water to make NCDs dispersion. Urea was held in a covered alumina crucible at 250°C for 1°h, 350°C for 1h, and 550°C for 2°h and calcined for 2 h at 600°C to make g-C_3_N_4_ nanosheets. The mixture of DI water, g-C_3_N_4_, AgNO_3_, and Na_2_HPO_4_ was centrifuged, washed with ethanol and DI water, vacuum-dried for 12 h at 45°C, and ground into powder to make g-C_3_N_4_/Ag_3_PO_4_. The mixture of g-C_3_N_4_/Ag_3_PO_4_ and NCDs was centrifuged, washed with ethanol and DI water, and vacuum-dried for 12 h at 45°C to make g-C_3_N_4_/Ag_3_PO_4_/NCDs. XRD pattern of g-C_3_N_4_/Ag_3_PO_4_/NCDs showed characteristic peaks of the cubic phase Ag_3_PO_4_. According to the TEM result, Ag_3_PO_4_ and NCDs particles were successfully loaded to g-C_3_N_4_. The XPS result revealed that g-C_3_N_4_/Ag_3_PO_4_/NCDs contained C, N, O, Ag, and P. The g-C_3_N_4_/Ag_3_PO_4_/NCDs photocatalyst was used for photodegradation of methylene blue (MB), rhodamine B (RhB), and phenol under visible light. 10 mg L^−1^ MB solution and 10 mg L^−1^RhB solution were degraded in 20 and 15 min, respectively, and 64% of 50 mg L^−1^ phenol solution was degraded in 80 min, much faster than other catalysts such as Ag_3_PO_4_ and g-C_3_N_4_/Ag_3_PO_4_. The g-C_3_N_4_/Ag_3_PO_4_/NCDs photocatalyst showed enhanced performance because NCDs have increased molecular oxygen activation, promoted charge transfer, and improved light absorption capacity. The photocatalyst could be used up to four times without a significant decrease in performance, suggesting that the photocatalyst has high stability.

Miao et al. developed an all-solid-state Z-scheme Ag_3_PO_4_/graphene oxide (GO)/nitrogen-doped carbon dot (NCD) photocatalyst by depositing NCDs on Ag_3_PO_4_/GO composite (Miao et al., 2018b). The mixture of water, citric acid, and ethylene diamine was held in a Teflon-lined autoclave for 5 h at 200°C, dialyzed for 1°day, freeze-dried, and dispersed in water to make NCDs dispersion. The mixture of DI water, GO, AgNO_3_, and Na_2_HPO_4_ was centrifuged, washed with ethanol and DI water, dried for 12 h at ambient temperature in the dark, and ground into powder to make Ag_3_PO_4_/GO. The mixture of DI water, Ag_3_PO_4_/GO, and NCDs was centrifuged, washed with ethanol and DI water, dried at ambient temperature in the dark, and ground into powder to make Ag_3_PO_4_/GO/NCD. The XRD pattern of Ag_3_PO_4_/GO/NCD exhibited peaks corresponding to the cubic phase Ag_3_PO_4_. From Raman spectroscopy, the D-band and G-band of carbon at 1,370 and 1,600 cm^−1^, respectively, and Ag_3_PO_4_ peaks at 905 and 1,000 cm^−1^ were observed. TEM images showed that Ag_3_PO_4_ and NCD particles were successfully incorporated with GO sheets. XPS result revealed that Ag_3_PO_4_/GO/NCD contained C, N, O, Ag, and P. According to photoluminescence (PL) spectra, Ag_3_PO_4_/GO/NCD exhibited lower PL intensity than its components. The Ag_3_PO_4_/GO/NCD photocatalyst was used for the degradation of methylene blue (MB), rhodamine B (RhB), and phenol under visible light. 10 mg L^−1^ MB solution, 10 mg L^−1^ RhB solution, and 50 mg L^−1^ phenol solution were degraded in 2.5, 5, and 120 min, respectively, which were much faster than those of Ag_3_PO_4_ and Ag_3_PO_4_/GO. The photocatalytic activity was improved because NCDs increased the oxygen activation, enhanced light absorption capacity, and suppressed photoinduced electron-hole pairs recombination. The Ag_3_PO_4_/GO/NCD photocatalyst could be used four times without a significant decrease in photocatalytic activity, showing the high stability of the photocatalyst.

Jana et al. prepared CuO-associated carbon dots (CDCs) in an eco-friendly way (Jana et al., 2020). The mixture of citric acid (CA), tetraethylenepentamine (TEPA), and copper sulfate pentahydrate was sonicated, heated in a microwave oven for 5 min, dissolved in water, centrifuged for 10 min at 10,000 rpm, and dialyzed for 12 h to make CDCs. The TEM result revealed that CDCs had an average size of 5.16 nm. XRD pattern of CDCs exhibited a broad peak of around 21° corresponding to amorphous carbon. Tge XPS result showed that CDCs contained C, N, O, and Cu. The zeta potential of CDCs was +5.42 mV, indicating that CDCs had a positive surface charge. CDCs were incorporated with a cationic dye rhodamine B (RhB), an anionic dye alizarin red S (ArS), or a neutral dye fluorescein C (FlC) into CDC-dye systems exhibiting photoelectric conversion processes. The CDC-RhB system displayed the lowest energy transfer efficiency (η) of 6.03% because the repulsion between positively charged CDCs and cationic RhB decreased the energy transfer. A positively charged surfactant cetrimonium bromide (CTAB) and a negatively charged surfactant sodium dodecyl sulfate (SDS) were used to enhance η of the CDC-RhB system, and the negatively charged surfactant enhanced η more, showing FRET efficiency $\eta_{FRET}$ of 14.7%.

Zhang et al. prepared a first-time novel synergistic catalyst (N-CDs/m-TiO_2_) by compounding m-TiO_2_ with N-CDs (Zhang and Dai, 2016). The mixture of citric acid (CA) Urea (U) and N-N-dimethylformamide (DMF) ultrasonication and held on PTFE autoclave in microwave vessel at 108°C for 20 min temperature elevating range of 15°C min^−1^, dialyzed for 48 h, freeze-dried this N-CDs/m-TiO_2_ were carried out for the preparation of N-CDs. The mixture of PS sphere and m-TiO_2_ heated at 70°C for 12 h in an oven further sintered motel furnace at a setting temperature of 500°C for 2 h for removing SP sphere to make macro porous TiO_2_(m-TiO_2_). The mixture N-CDs and m-TiO_2_ was transferred into the heating mantle at a set temperature of 60°C for 12 h by the thermal deposition technique. XRD of N-CDs/m-TiO_2_ exhibited peaks at 25.3°, 48.1°, 53.8°, 55.2°, and 62.8°, corresponding to (101), (200), (105), (211), and (204), respectively for the anatase TiO_2_. For the FTIR spectra of N-CDs/m-TiO_2_, the additional peak appears at 1,050 cm^−1^, corresponding to bending vibration of C-NH-C, and the absorption peak appears between 3,600 and 3,300 cm^−1^ for the TiO_2_ NPs and m-TiO_2_ for a fact at N-CDs use capable of enhancing charge transfer rate as well as CQDs beneficial for promotion photocatalytic efficiency of TiO_2_. The N_2_ adsorption-desorption isotherm of N-CDs/m-TiO_2_ demonstrates the hysteresis loops at relative pressure 0.2 with type IV adsorption-desorption having uniform pore size distribution. For the UV-DRS spectra of N-CDs/m-TiO_2_, the threshold wavelength (λ_g_) of N-CDs/m-TiO_2_ was 511.49 nm and band gap energy (Eg) was 2.42 eV. The N-CDs/m-TiO_2_ shows the higher photo-anode, photocurrent intensity. There was a higher photochemical activity of N-CDs/m-TiO_2_ due to its unique porous structure and sensitization of N-CDs. The N-CDs/m-TiO_2_ shows excellent photovoltaic conversion efficiency and stability.

The photocatalytic performance of N-CDs/m-TiO_2_ was examined by using MB as a target pollutant under UV light irradiation. For N-CDs/m-TiO_2_ to enhanced degradation efficiency, the decolorization ratio at MB over N-CDs/m-TiO_2_ still shows excellent recycling ability of the N-CDs/m-TiO_2_ photocatalyst. During photocatalytic degradation of MB to determine the major oxidant, that is, ethylene diamine tetra-acetic acid (EDTA), tert-butanol (TBA), and p-benzoquinone (BQ) were employed as h^+^, OH·, and O_2_ scavengers, respectively. The luminescence property of N-CDs/m-TiO_2_ significantly enhanced photocatalytic performance due to the synergistic effect between N-CDs sensitization and the unique porous structure of m-TiO_2_. In MB containing aromatic ring is responsible for the π-π conjugated structure with the π orbitals of N-CDs, and hence enhancing the photocatalytic activity of N-CDs/m-TiO_2_ on the MB surface.

**FIGURE 9:**
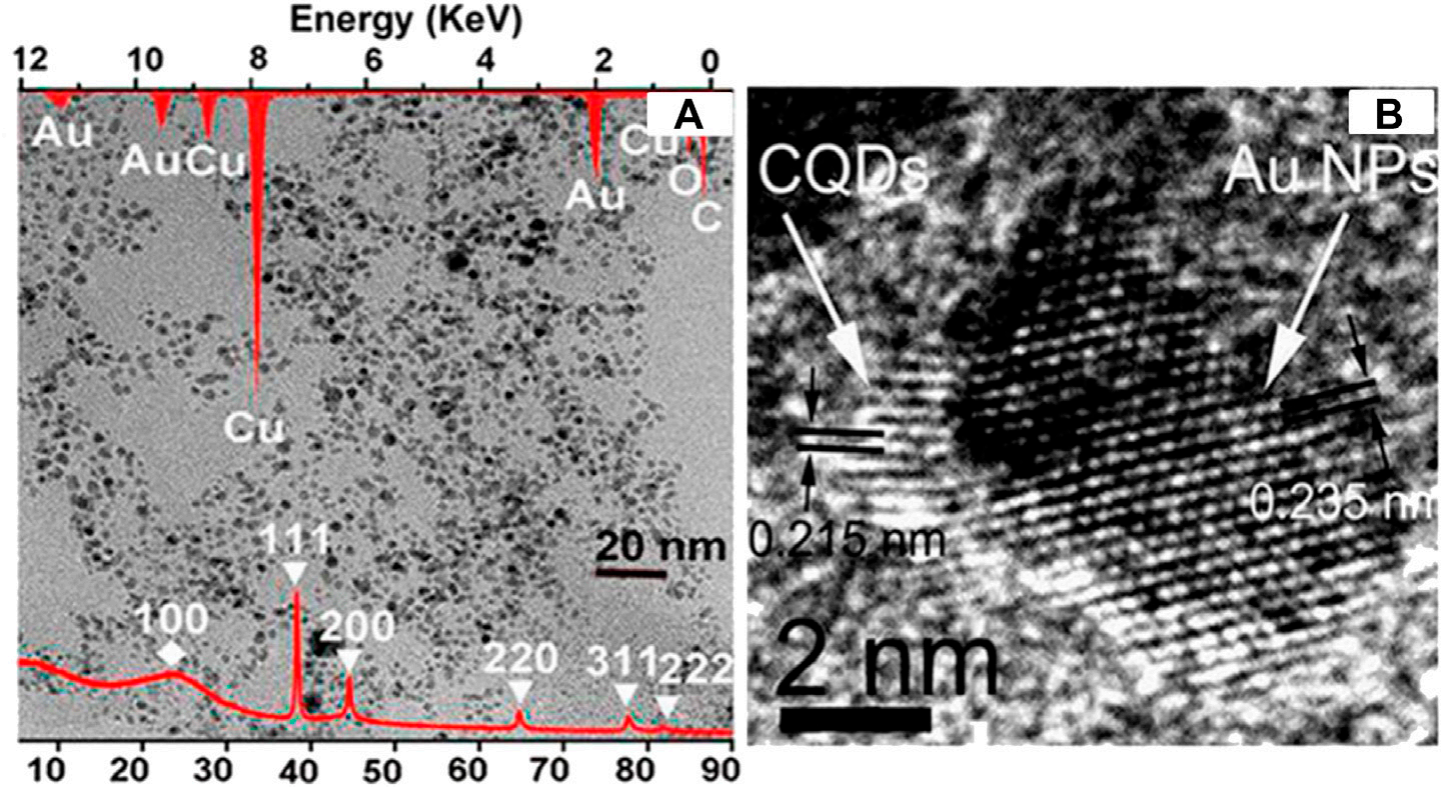
**(A)** Transmission electron microscopy (TEM) image, X-ray diffraction (XRD) pattern (shown as the red overlay at the top portion of the panel) and energy-dispersive X-ray (EDX) spectrum (shown as the red overlay at the bottom portion of the panel) of Au/CQDs composites; **(B)** high-resolution transmission electron microscopy (HTEM) image of Au/CQDs composites. (Reproduced with permission from Liu et al., 2014, Copyright © 2014 American Chemical Society).

Li et al. synthesized carbon dots modified g-C_3_N_4_/SnO_2_ photocatalyst (CDs/g-C_3_N_4_/SnO_4_) using the thermal polymerization method (Li et al., 2021). Preparation of g-C_3_N_4_ using guanidinium hydrochloride heated in a muffle furnace at 550°C for 12 h with a ramp rate of 2°C min^−1^. Herein to make SnO_2_ nanoparticles; SnCl_4_.H_2_O dissolved in DI water followed with 17 h ultrasonication and the mixture is transferred into stainless steel autoclave and processed further at 180°C for 12 h. The mixture further rinsed with ethanol and DI water dried at 60°C for 24 h. The mixture of CN and SnO_2_ was dissolved in ethanol heated at 60°C to remove the ethanol mixture calcined at 400°C for 2 h with a ramp rate of 2°C min^−1^. CDs were prepared by using a mixture of citric acid (CA), urea (U), and DI water held in a Teflon-lined stainless steel autoclave at 180°C for 5 h. CDs/CN/SnO_2_ was prepared using CSn_2_0 and CDs with ethanol heated at 60°C for evaporating ethanol and then heated at 300°C for 3 h. CDs/CN (CCN) and CDs/SnO_2_ (CSnO_2_) were prepared by the same method to make 0.5 CCSn20. The TEM, HTEM, and FPT pattern clearly shows the hexagonal crystalline structure of CDs (Figures 9B,C), confirming that the CDs dispersed in g-C_3_N_4_ and SnO_2_ assigned (110) planes. XRD spectra of 0.5 CCSn20 show that a high purity crystalline state of materials was synthesized. The N_2_ adsorption-desorption isotherms of 0.5 CCSn20 photocatalysts showed a higher specific surface area (53.717°m^2^/g). XPS spectra reveal that 0.5 CCSn20 contains C, N, O, and Sn3d. UV-vis DRS for 0.5 CCSn20 increased light absorption capacity more than its components. According to photoluminescence (PL) spectra, 0.5 CCSn20 exhibited lower PL intensity than its components. The 0.5 CCSn20 photocatalyst was used for the degradation of indomethacin (IDM) under visible light. The photocatalytic IDM degradation rate of 0.5 CCSn20 was higher by 70.3% than CN (32.0%).

**FIGURE 10:**
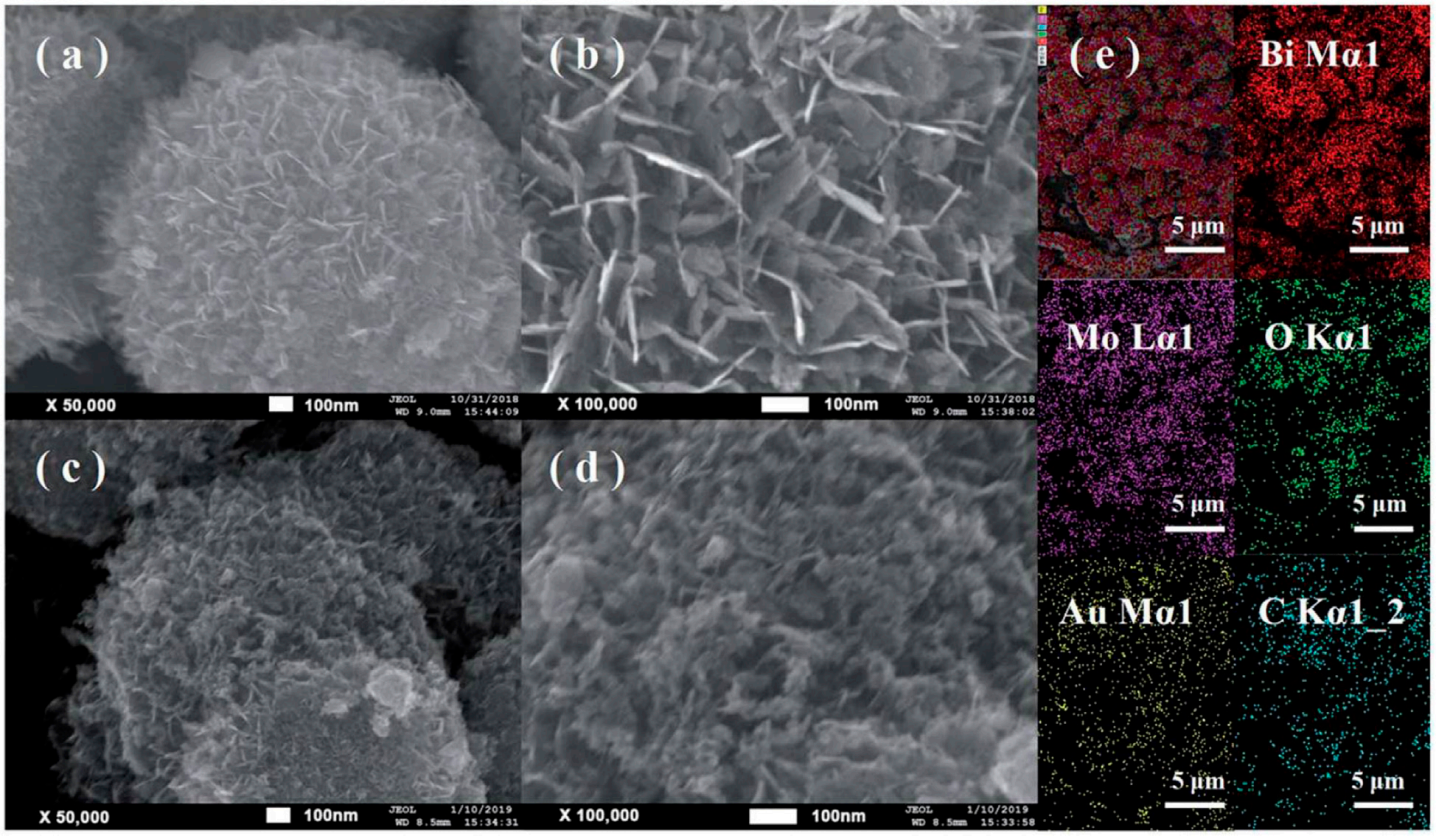
FE-SEM images of **(A,B)** hollow BMO microsphere and **(C,D)** CQDs/Au/BMO; **(E)** EDX spectrum of sample CQDs/Au/BMO showing the presence of Bi, Mo, O, Au and C elements. (Reproduced from Zhao et al., 2021, Royal Society of Chemistry).

Yu et al. prepared ZnO/CQDs nanocomposites for the one-step hydrothermal reaction method (Yu et al., 2012). The mixture of Zn (AC_2_) 2H_2_O and the alcoholic solution of CQDs was held in a Teflon-lined stainless steel autoclave heated at 100°C for 8 h washed with DI water and ethanol and then vacuum-dried at 60°C for 24 h. XRD of ZnO/CQDs shows the successful formation of ZnO/CQDs diffraction peak at ZnO corresponding to wurtzite ZnO phase (JCPDS-36-1451). Raman spectrum shows prepared ZnO/CQDs nanocomposites are composed at CQDs and ZnO. FTIR spectra results showed CQDs are successfully attached to ZnO nanoparticles. UV-Vis ZnO/CQDs nanocomposites showed higher photocatalytic activity than other components. SEM showed small uniform nanoparticles with a size of 20–30 nm of ZnO/CQDs. HTEM and TEM of ZnO/CQDs formation of carbon layer due to the addition of CQDs are shown in Figures 10A,B. EDS gives the elements present in ZnO/CQDs nanocomposites Zn, O, and C. The ZnO/CQDs nanocomposite was used as the photocatalytic ability for photodegradation of gas-phase benzene under visible light carried out at room temperature. The photocatalytic degradation efficiency of gas-phase benzene and gas-phase methanol was found at 86% and 82% using ZnO/CQDs higher than that of ZnO at 26%, 22%, and N-doped TiO_2_ (N–TiO_2_) at 60% and 55%, respectively. The CODs were loaded on the ZnO surface to the main function for the enhanced photocatalytic activity of ZnO/CQDs because ZnO/CQDs had better performance than ZnO due to CQDs increased photocatalytic activity toward degradation of toxic gas (benzene and methanol) under visible light at RT.

Nie et al. synthesized CDs/NiCo_2_O_4_ photocatalyst (Nie et al., 2021). Nickel nitrate, cobalt nitrate, and DI water were stirred; CDs were added in different wt% into the mixture, sonicated, and stirred, and then the solution was moved to porcelain crucible. Mixtures of the solution were then heated at 400°C for 2 h in a muffle furnace, washed with water and ethanol, and then dried at 70°C in a vacuum oven to make CDs/NiCo_2_O_4_. TEM and H-TEM images reveal that the CDs/NiCo_2_O_4_ exhibits irregular spherical shapes, and the interplanar spacing of 0.21 and 0.24 nm is associated with the (100) spacing of CDs and (311) spacing of NiCo_2_O_4_ shown in Figures 10C,D. The XRD pattern of CDs/NiCo_2_O_4_ perfectly matches with (JCPDS No. 73-1702) card. XPS result confirmed that CDs/NiCo_2_O_4_ nanocomposite contained Ni, Co, C, and O. Electrochemical band structure of the CDs/NiCo_2_O_4_ photocatalyst reveals that helps photocatalytic overall water decomposition. According to TPV, the measurement of CDs/NiCo_2_O_4_ showed greater photocurrent intensity, and *in situ* TPV measurement study during the reaction of photocatalytic HER and OER further enhanced the reaction rate after being supported with CDs. The EIS spectra of CDs/NiCo_2_O_4_ demonstrates is having lower charge transfer resistance, and hence it showed better photocurrent intensity, and higher overall water decomposition reaction activity. The CDs/NiCo_2_O_4_ showed the best possible hydrogen (oxygen evolution) production is up to 62 μmol h^−1^ g^−1^ (29 μmol h^−1^ g^−1^). The CDs/NiCo_2_O_4_ is still stable toward H_2_ production. According to the TPV test, the CDs enhanced the interface electron extraction by about 0.09 ms, while the maximum electron storage time by CDs is up to 0.7 ms, concluding that CDs enhanced the photocatalytic water decomposition activity.

Liu et al. prepared metal nanoparticles/carbon quantum dot composite photocatalyst using Au and CQDs (Liu et al., 2014). CQDs were prepared by electrochemical ablation of graphite. Two graphite rods were placed in an ultrapure water electrolyte solution, applying static potential 15–60 V by both electrodes using direct current for 10 days, with stirring then centrifuged to make CODs. HAuCl2 and CQDs solution stirred dark environments at room temperature for 2 h overnight, centrifuged, washed DI water, and ethanol-dried for 6 h at 25–60°C to make Au/CQDs. EDX results demonstrated the elemental composition of Au, C, and O from AuNPs and CQDs. XRD pattern of Au/CQDs exhibited planes (111), (200), (220), (311), and (222) for AuNPs and a strong peak at 20° for carbon. The TEM image showed the successful formation of Au/CQDs (Figure 9). UV-Vis absorption spectra of Au/CQDs showed both UV-Vis absorption peaks at 230 nm and 490–590 nm for CQDs and AuNPs, respectively. Raman of Au/CQDs composites showed a higher I_D_/I_G_ ratio than AuNPs and CQDs. Photocatalytic oxidation of cyclohexane to cyclohexanone with Au/CQDs composites under visible light is increased could be due to a similar combination and interaction of CQDs and AuNPs. According to PL spectra, CQDs were excellent, as they were quenched by either an electron donor or an electron acceptor. Electrocatalytic studies on Au/CODs composite toward hydrogen peroxide decomposition shows excellent activity under visible-light irradiation than that absence of light irradiation. Au/CQDs composite exhibited high photocatalytic activity selective for oxidation of cyclohexane to cyclohexanone using hydrogen peroxide as oxidant solvent-free. Electrochemical study reveals that visible light enhanced catalytic reaction.

Liang et al. prepared ZnO sensitized by carbon quantum dots (L-CQDs/ZnO) by a combination of L-CQDs and ZnO using the hydrothermal method (Liang et al., 2020). Zinc oxide, ammonia water, and ammonium bicarbonate were mixed together, filtered, and stirred for 4 h. The supernatant was steamed at 90 ± 2°C, filtrated, washed, dried in a vacuum oven at 120 ± 2°C, and then annealed at 500 ± 2°C for 4 h to make ZnO. ZnO was added into 120 ml L-CQDs aqueous solutions, stirred for 2 h, held into stainless steel autoclave heated air dry oven at 120 ± 2°C for 2 h, cooled, filtrated, washed with water dried at 60 ± 2°C for 12 h, and dried at vacuum oven to make L-CQDs/ZnO. TEM result demonstrated that the L-CQD shad average diameter of 2.1 ± 0.4 nm (Figures 12A,B). FTIR spectra demonstrated the strong absorption peak at 400–800 cm^−1^ of L-CQDs/ZnO, which confirmed strong physical interaction between carbon and zinc atoms. According to XPS results, L-CQDs/ZnO contained Zn, C, O with peaks appears at 1,021.1 eV (Zn_2_p_3/2_) and 1,044.2 eV (Zn_2_p_1/2_), 284.8 eV, 288.3 eV (C1s) and 531.8 eV, 532.6 eV (O1s). UV-Vis DRS results demonstrated that the L-CQDs/ZnO showed better photocatalytic activity. According to PL results, the L-CQDs/ZnO had lower photogenerated electron-hole pair recombination. The result demonstrated the efficient electron transport caused by the π bond conjugate system of L-CQDs. The L-CQDs/ZnO was used as a photocatalyst for the degradation of phenol under sunlight. The L-CQDs/ZnO’s highest degradation efficiency (60%) completely degrades phenol at 5.5 h compared to ZnO. The L-CQDs/ZnO exhibited better degradation efficiency than ZnO because wt% of CQDs increased charge separation efficiency.

Bozetine et al. developed ZnO/CQDs/AgNPs by the green approach of the *in situ* hydrothermal method (Bozetine et al., 2021). d-Lactose source of carbon and NaOH solution held in Teflon-beaker heated at 80°C for 15 min for the synthesis of CQD. Further to this CQD dispersion added Zn(CH_3_COO)_2_ at RT and adjusted pH-10 by addition of 8 M NaOH dropwise with constant stirring for 15 min. The mixture was heated at 80°C for 2 h and added drop-wise silver nitrate under stirring at 80°C for 1 h to make ZnO/CQDs/AgNPs. The XRD pattern of ZnO/CQDs/AgNPs exhibited a peak at 30°, 44.5°, and 64.6°, corresponding to (111), (200), and (220) planes Ag. According to SEM results, ZnO/CQDs/AgNPs had particles size (<50 nm). The EDX results demonstrated that chemical composition consisted of Zn, C, O, and Ag elements. XPS results demonstrated that ZnO/CQDs/AgNPs contained Zn, O, C, and Ag. N_2_ adsorption–desorption isotherms of ZnO/CQDs/AgNPs ternary achieved the highest surface area compared to the composite of ZnO, Ag, and binary of CQDs. UV-Vis spectra of ZnO/CQDs/AgNPs achieved band gap at 3.22 eV to generate electron-hole pairs. The ZnO/CQDs/AgNPs composite was used for photocatalysis of MB dye under UV-light irradiation. The photodegradation efficiency of MB dye was 98.6% for ZnO/CQDs/AgNPs. ZnO/CQDs/AgNPs photocatalyst achieved higher photocatalytic activity under UV-light irradiation due to the AgNPs, and CQDs reduced the recombination rate of the photogenerated electron-hole pairs and enhanced photocatalytic reactions.

**FIGURE 11:**
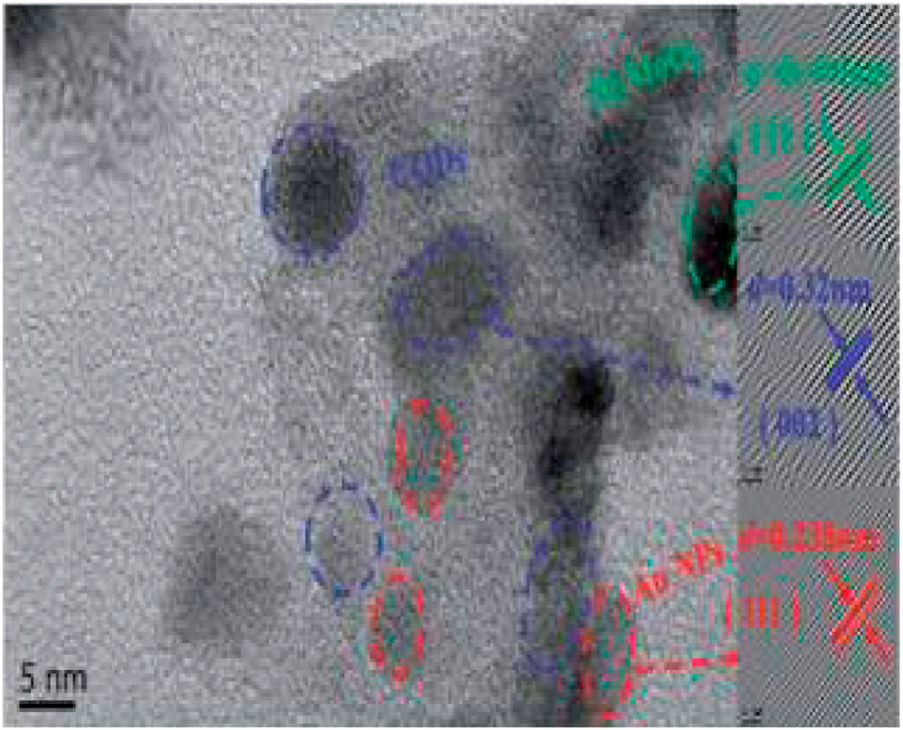
HTEM image of CQDs/Au/BMO nanocomposite. (Reproduced from Zhao et al., 2021, Royal Society of Chemistry).

Zhao et al. synthesized carbon quantum dots and Au nanoparticles with Bi_2_MoO_6_ by simple hydrothermal method (CQDs/Au/BMO) (Zhao et al., 2021). Citric acid and urea were held in a Teflon-lined autoclave for 5 h at 180°C, centrifuged for 30 min, and subjected to vacuum drying for 3 h at 80°C to make CQDs. Bi(NO_3_)_3_ 5H_2_O and Na_2_MoO_4_ 2H_2_O were added in ethylene glycol, and ethanol stirring was held in an autoclave for 12 h at 2016°C, centrifuged, washed with water and alcohol, and dried overnight in an electric oven at 60°C to make BMO. BMO, HAuCl_4_, and lysine were added in DI water, maintained pH with added NaOH and NaBH_4_, centrifuged, washed with water, and dried overnight in an oven at 60°C to make Au/BMO. CDs and Au/BMO were dispersed in ethanol using ultrasonic treatment followed by drying and calcination 2 h at 250°C to make CQDs/Au/BMO. XRD pattern of CQDs/Au/BMO showed peaks at 38.2° and 44.3° corresponding to (111) and (200) planes for Au NPs for Au/BMO and CQDs/Au/BMO. The XPS result demonstrated that CQDs/Au/BMO contained Bi, Mo, O, Au, and C elements. The morphology and EDX of BMO possesses a hierarchical microsphere with an average diameter was about 1–2 mm, CQDs/Au/BMO are similar to that of BMO, EDX indicated that all the elements are uniformly distributed (Figures 10A–E). According to the H-TEM result, CQDs/Au/BMO revealed (131), (002), and (111) planes for BMO, CQDs, and Au, respectively (Figure 11). EPR spectra showed the oxygen vacancies in BMO and CQDs/Au/BMO. The CQDs/Au/BMO was used as a photocatalyst for the degradation of phenol under visible light. The phenol degradation rate constant of 7% CQDs/Au/BMO was 228.21 × 10^−4^ min^−1^. The 7% CQDs/Au/BMO exhibited the highest phenol degradation efficiency of 94%, which was higher than that of BMO-SOVs (24%), Au/BMO (45%), and CQDs/BMO (51%). According to PL and EIS spectra, 7% CQDs/Au/BMO showed higher photocatalytic activity due to the interaction of CQDs and Au NPs on the surface of BMO. The EIS result confirmed that CQDs/Au/BMO showed the strongest ability to separate and transfer photogenerated e^−^/h^+^ and enhanced photocatalytic activity.

Hu et al. developed carbon dots-decorated BiOCOOH/ultrathin g-C_3_N_4_ nanosheets (CQDs/BiOCOOH/uCN) photocatalyst by the simple method (Hu et al., 2021). Melamine was calcined for 4 h at 550°C to make bulk g-C_3_N_4_. The bulk g-C_3_N_4_ was held in an aluminum crucible and thermally exploited in uCN for 2 h at 500°C to make uCN. Citric acid and ethylene diamine were held in a Teflon-lined autoclave for 5 h at 200°C and dialyzed for 24 h to make CQDs. The mixture of uCN, HCOONa and Bi (NO_3_)_3_·5H_2_O is taken in water and stirred for 30 min followed by added CQDs solution. Furthermore the solution was transfer into Teflon lined autoclave and heated at 150°C for 10 h to make CQDs/BiOCOOH/uCN. The SEM image of CQDs/BiOCOOH/uCN showed a flower-like structure. EDS of CQDs/BiOCOOH/uCN contained Bi, O, N, and C elements. HTEM of CQDs/BiOCOOH/uCN demonstrated that the lattice stripes and planes ((102) (110)), (101), and (002)) for BiOCOOH, graphitic carbon, and uCN.XRD pattern of CQDs/BiOCOOH/uCN exhibited peaks at 13.1° and 27.4°, corresponding to (100) and (002) for uCN and board peak 25° for the disorder of carbon. XPS spectra revealed that CQDs/BiOCOOH/uCN contained C, N, Bi, and O elements. The photoluminescence (PL) spectra showed that CQDs/BiOCOOH/uCN exhibited the strongest emission when the excitation wavelength was 460 nm. The CQDs/BiOCOOH/uCN was used as a photocatalyst for the degradation of sulfathiazole (STZ) under LED lamp irradiation. Using the CQDs/BiOCOOH/uCN showed higher photocatalytic activity compared to its composites. The photocatalytic effect was improved because of the formation of heterojunction. Photodegradation efficiencies of STZ at various pH values were studied up to 98%, and the 4-CQDs/BiOCOOH/uCN showed excellent photocatalytic activity, both acidic and alkaline. The degradation efficiency of STZ was checked by adding natural organic matter and inorganic anions, which revealed CQDs/BiOCOOH/uCN was an attractive material for both NOM and IA. The CQDs/BiOCOOH/uCN showed better photocatalyst activity.

Gao et al. prepared N-CQDs/BiOI_x_Br_1-x_ by the one-step *in situ* co-precipitation method (Gao et al., 2021). Citric acid and urea ultrasonication 2 h were mixture held in a Teflon-lined stainless steel autoclave for 5 h at 180°C, dialyzed for 24 h, and dried for 12 h at 60°C to make N-CQDs. The Bi(NO_3_)_3_⋅5H_2_O in glycol is ultrasonicated for better dispersion and added N-CQDs under stirring for next 2 h to make N-CQDs/BiOI_x_Br_1−x_. The XRD spectra of N-CQDs/BiOI_x_Br_1-x_ exhibited a characteristic peak at 2θ = 26° to 35°. According to SEM images, the N-CQDs/BiOI_x_Br_1-x_ were nanosheets thickness of about 23 nm. HTEM results demonstrated the presence of lattice fringes and an average diameter of about 7 nm. XPS result demonstrated that N-CQDs/BiOIxBr1-x contained Bi, O, I, Br, C, and N elements. According to the Brunauer–Emmett–Teller (BET) analysis of N-CQDs/BiOIxBr1-x, the large specific surface area was 20.97 m^2^/g. The N-CQDs/BiOIxBr1-x was used as a photocatalyst for the degradation of phenol under visible light irradiation. The photocatalytic phenol degradation rate of N-CQDs/BiOIxBr1-x was higher 98.7% than its composites. The enhancement of the photocatalytic activity of N-CQDs/BiOIxBr1-x was due to the scattering of light and layered BiOxBr1-x, as well as the internal electric field from N-CQD to BiOxBr1-x.

## 5 Conclusion

After CQDs were discovered in 2004, there have been many investigations on their synthesis, properties, modifications, and applications due to their distinctive optical and electronic properties. This work focuses on recent progress on CQDs-related materials’ synthesis, properties, and applications in photocatalysis. Many simple, low-cost, and scalable synthesis routes of CQDs-related materials have been devised. CQDs exhibited excellent sunlight harvesting ability, tunable PL, UCPL, and efficient photoexcited electron transfer. CQDs can act as a sole photocatalyst or enhance the photocatalytic activity of other photocatalysts as an electron mediator, a photosensitizer, and/or a spectral converter.

Despite significant improvement over the previous decade, various issues still require much investigation. Defects in CQDs modify their optical and electrical characteristics dramatically. Despite this, there are no established methodologies or tactics for dealing with CQD issues. Further research on CQD structure synthesis using dense and precise stages is required. CQD modification has sparked much interest in the scientific community. Many applications can benefit from better PL control and efficiency by readily functionalizing and doping CQDs. In the future, the mechanisms of CQD-associated phenotypes will be investigated. The UCPL mechanism of CQDs, for example, is currently unknown. As a result, additional research is needed to determine the photogenerated electron transfer route in the UCPL process. Due to UV absorption, photocatalytic CQDs have a low light-harvesting capability. CQDs that can be triggered in the visible or even in the near-infrared range can be created. This will aid heterogeneous photocatalysts in making use of the solar spectrum. Few studies have been conducted on the photostability of CQD-based nanocomposites, which are difficult to fabricate. The presence of oxygen groups on CQD surfaces may affect photocatalytic activity. The loss of oxygenic functional groups during photocatalysis doubts the photostability of CQD-based photocatalysts. As a result, CQDs will play a significant role in future photocatalytic devices. Innovative, cost-effective, and simple synthetic methodologies, as well as innovative photocatalytic applications for CQDs, will be developed.
